# Mental health impacts experienced by caregivers of people with Dravet syndrome: A systematic literature review

**DOI:** 10.1002/epi.70171

**Published:** 2026-03-30

**Authors:** Adam Strzelczyk, Mary Anne Meskis, Galia Wilson, Bobby Jacob, Christoph Helmstaedter, Jane von Gaudecker, Veronica Hood, Ceri Hughes, Michael Scott Perry

**Affiliations:** ^1^ Epilepsy Center Frankfurt Rhine‐Main, Department of Neurology, University Medicine Frankfurt Goethe University Frankfurt Frankfurt am Main Germany; ^2^ Dravet Syndrome Foundation Cherry Hill New Jersey USA; ^3^ Dravet Syndrome UK Chesterfield UK; ^4^ UCB Smyrna Georgia USA; ^5^ Department of Epileptology University Clinic Bonn Bonn Germany; ^6^ Indiana University Indianapolis Indiana USA; ^7^ Cook Children's Medical Center Fort Worth Texas USA

**Keywords:** anxiety, depression, fatigue, isolation, sleep quality

## Abstract

**Objective:**

Dravet syndrome (DS) places tremendous burden on caregivers owing to the extent of required assistance and impact on daily living, as well as the risk to the individual with DS of premature mortality from sudden unexpected death in epilepsy and morbidity associated with nonseizure manifestations. This systematic literature review provides an up‐to‐date characterization of the mental health impacts experienced by caregivers of people with DS.

**Methods:**

Databases (1974 to August 29, 2024 in Embase; 1946 to August 29, 2024 in MEDLINE) were searched for records containing keywords relevant to mental health in caregivers of people with DS. The study population comprised caregivers of people with DS with any or no intervention and/or comparator (and excluding pharmacologic interventions affecting caregiver burden‐related outcomes) and with mental health outcomes that included depression, anxiety, fatigue, sleep quality, stress, mood, and quality of life scales.

**Results:**

Database searches returned 519 records; 20 published articles were included. Most common were cross‐sectional studies, with populations from Asia, Australia, Central/South America, Europe, and North America. Study sample sizes ranged from seven to 256 caregivers of people with DS; most caregivers were female. Depression and anxiety were reported in 11 and 10 articles, respectively; the prevalence of depression and anxiety among caregivers ranged 5%–66% and 5.2%–80%, respectively. Some studies used instruments to assess mental health outcomes; Beck Depression Inventory‐II for depressive symptoms and the Hospital Anxiety and Depression Scale for symptoms of anxiety and depression were reported in three and two articles, respectively. Factors potentially associated with mental health including sleep quality, fatigue, and stress were commonly reported, with poor sleep quality and fatigue often linked to nighttime monitoring of people with DS.

**Significance:**

Physicians should routinely assess the mental health of caregivers of people with DS; future studies should focus on identifying interventions that ease burden on caregivers.

## Graphical abstract

Graphical abstract for the systematic literature review.

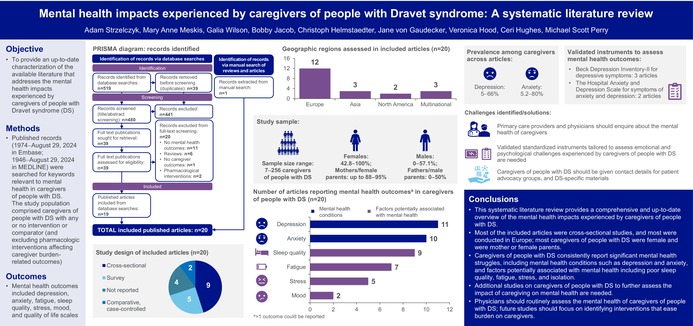

**Key points**

- DS places a heavy burden on caregivers, given the assistance patients require and impact on caregivers' daily lives.
- Of 519 records identified, 20 published articles were included; sample size ranged from seven to 256 caregivers of people with DS, mostly females.
- Caregivers reported mental health impacts (depression and anxiety) and related factors such as poor sleep quality, fatigue, and stress.
- Validated standardized instruments to assess emotional and psychological challenges faced by caregivers of people with DS are needed.
- Physicians should assess the mental health of caregivers of people with DS; interventions that ease caregiver burden should be identified.

## 1 INTRODUCTION

Dravet syndrome (DS) is a rare, drug‐resistant, childhood onset developmental epileptic encephalopathy characterized by the onset of seizures before 20 months of age.^1^ DS was first described in 1978 by Dravet^2^ and originally named severe myoclonic epilepsy of infancy. The condition has since been renamed DS, given this rare neurological condition is not limited to childhood but persists into adulthood,^3^ and it has a distinct pattern with increasing comorbidities with age.^4^, ^5^ There are few epidemiological studies on DS.^6^ A systematic review of the literature from inception to June 2022 reported the incidence of DS in the United States and Europe ranged from 1/15 400 to 1/40 900, and the prevalence ranged from 1.5/100 000 to 6.5/100 000.^1^

DS is characterized by prolonged, recurrent seizures^3^ and the development of nonseizure manifestations, which include cognitive, motor, and behavioral problems.^7^ The risk of premature mortality from sudden unexpected death in epilepsy (SUDEP) or status epilepticus, the change in seizures types over time, and the morbidity associated with nonseizure manifestations all contribute to the humanistic and economic burden of the disease and negatively impact health‐related quality of life (HRQoL).^8^ In turn, the clinical manifestations and comorbidities associated with DS affect caregivers (individuals such as family and non‐family members who care for people with DS, and help address the significant and unique needs of people with DS), owing to the extent of patient assistance needed and the impact on caregivers' daily lives.^8^ The objective of this systematic literature review (SLR) is to provide an up‐to‐date characterization of the available literature that addresses the mental health impacts experienced by caregivers of people with DS.

## 2 MATERIALS AND METHODS

This SLR was conducted in accordance with the Preferred Reporting Items for Systematic Reviews and Meta‐Analyses (PRISMA) 2020 statement^9^ and the PRISMA Protocol guidelines,^10^ and it was registered in the International Prospective Register of Systematic Reviews (PROSPERO; CRD42024605221) to avoid duplication and reduce potential reporting bias.

Caregivers were defined as those who provide care to people with DS who need assistance with activities of daily living because of illness, injury, or disability. Caregivers included family members such as parents and siblings, as well as non‐family members such as neighbors and friends. Paid professionals were not included. The location of residence of people with DS was not assessed.

Embase and MEDLINE (including records classified as ePublication ahead of print and in‐process, in‐data‐review, and other nonindexed citations) were rigorously and objectively searched via the Ovid interface for records containing keywords relevant to mental health in caregivers of people with DS (or severe myoclonic epilepsy of infancy; Table S1). Published records from 1974 to August 29, 2024 were searched in the Embase database, and from 1946 to August 29, 2024 in the MEDLINE database (MEDALL: Ovid MEDLINE ALL).

Included studies were defined according to the population, intervention, comparison, outcome, and study design framework; inclusion and exclusion criteria are reported in Table 1. Mental health outcomes were categorized into mental health conditions (depression and anxiety) and factors potentially associated with mental health (fatigue, sleep quality, stress, and mood). Studies included in this SLR reported depression and anxiety as symptoms rather than as clinical diagnoses.

**TABLE 1:** Study inclusion criteria.

	Inclusion criteria	Exclusion criteria
Population	Caregivers of people with DS	People with DS Paid caregivers of people with DS
Intervention/comparator	Any or none	Pharmacological interventions affecting outcomes related to caregiver burden
Outcomes	Mental health outcomes including but not limited to: Depression Anxiety Fatigue Sleep quality Stress Mood QoL scales	Economic burden Work productivity
Study and publication types	Randomized and nonrandomized controlled trials Observational studies and real‐world evidence	Preclinical, animal, pilot, or phase 1 clinical studies, protocols (without results), case reports/studies, case series Notes, commentaries, letters, editorials, opinions Meta‐analyses, reviews^a^ Conference abstracts
Language	No restriction	
Time	No restriction	

Abbreviations: DS, Dravet syndrome; QoL, quality of life.

^a^ Reviews were excluded, but reference lists for relevant systematic reviews were screened for primary sources.

Records returned from database searches were downloaded to EndNote bibliographic software, with duplications removed manually using EndNote algorithms. Duplicate citations were retained for transparency. Prescreening excluded records that did not meet the inclusion criteria. Abstract and title screening of records was performed by a reviewer, and a second reviewer screened 20% of the records. If there were discrepancies, reviewers discussed their reasoning until consensus was reached; otherwise, references were included in full‐text screening. Full‐text articles were obtained for citations that met inclusion criteria. A reviewer performed full‐text screening of records and provided reasons for exclusion. Discrepancies were discussed by two researchers or by consulting a third researcher. Reference lists from included reviews and articles were manually searched for publications that may not have appeared in the database searches.

The Mixed Methods Appraisal Tool (MMAT), version 2018,^11^ was used to assess included publications for quality/risk of bias (Table S2). A researcher assessed the quality of each study, which was checked by a second researcher. Discrepancies were discussed by two researchers or by consulting a third researcher.

After selection of publications based on full‐text screening, data extraction tables (DETs) were developed in a Word document. DETs were designed to capture study and population details as well as outcomes per the eligibility criteria. Data entered in the DETs were checked for accuracy by a second reviewer.

## 3 RESULTS

### 3.1 Records identified

**FIGURE 1:**
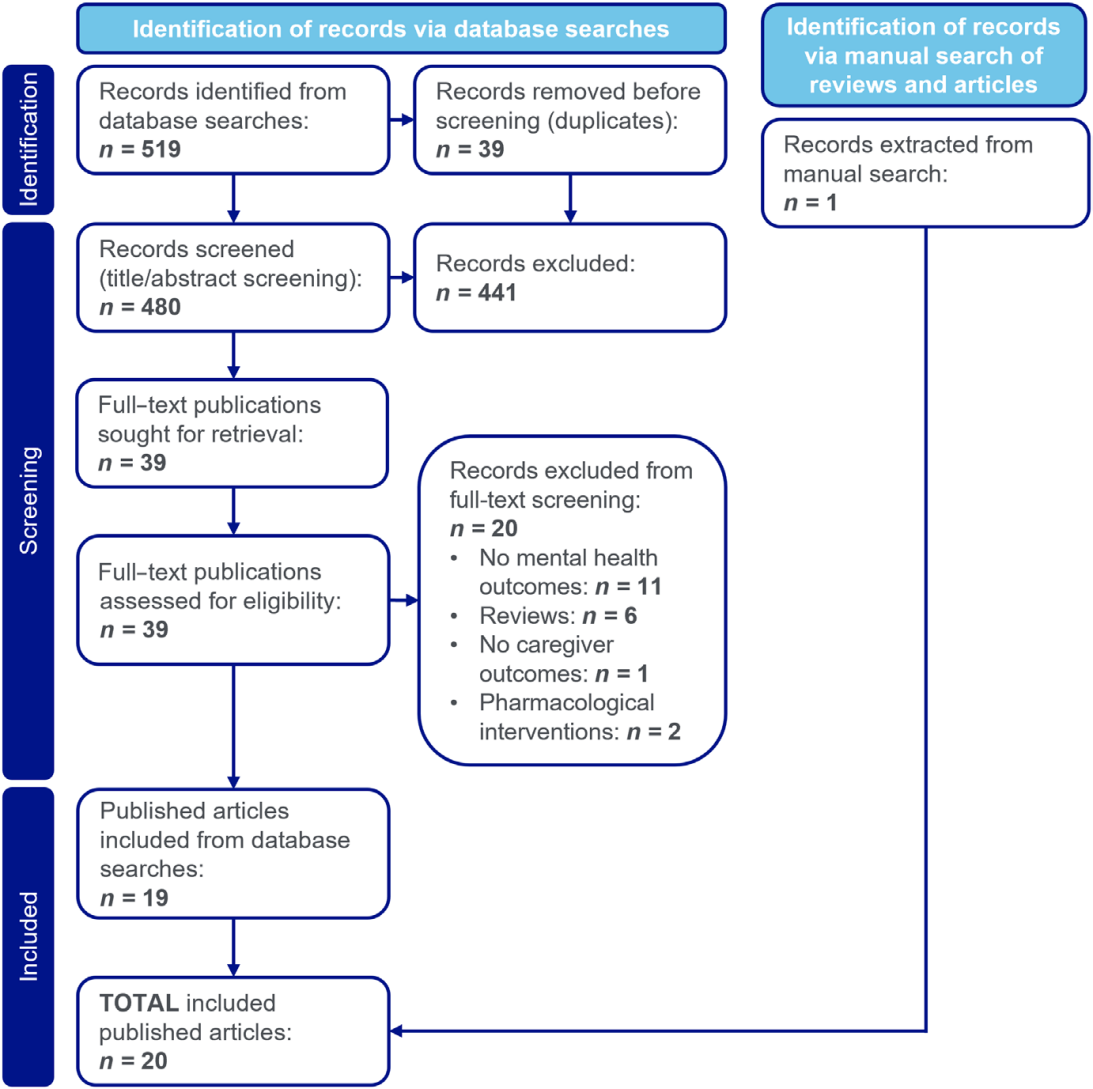
PRISMA (Preferred Reporting Items for Systematic Reviews and Meta‐Analyses) diagram: systematic literature review publication selection.

A total of 519 records were identified, of which 39 duplicates were excluded (Figure 1); 480 records underwent title and abstract screening, of which 441 records were excluded, and 39 full‐text publications were retrieved. In total, 20 published articles were excluded at the full‐text screening step (Table S3), and 20 full‐text published articles^4^, ^12^, ^13^, ^14^, ^15^, ^16^, ^17^, ^18^, ^19^, ^20^, ^21^, ^22^, ^23^, ^24^, ^25^, ^26^, ^27^, ^28^, ^29^, ^30^ were included: 19 from the database searches^4^, ^12^, ^13^, ^14^, ^15^, ^16^, ^17^, ^18^, ^19^, ^20^, ^21^, ^22^, ^23^, ^24^, ^25^, ^26^, ^27^, ^28^, ^29^ and one from the reference list search.^30^ For each of the included 20 articles, MMAT results for quality/risk bias are reported (Table S4).

### 3.2 Study characteristics

**FIGURE 2:**
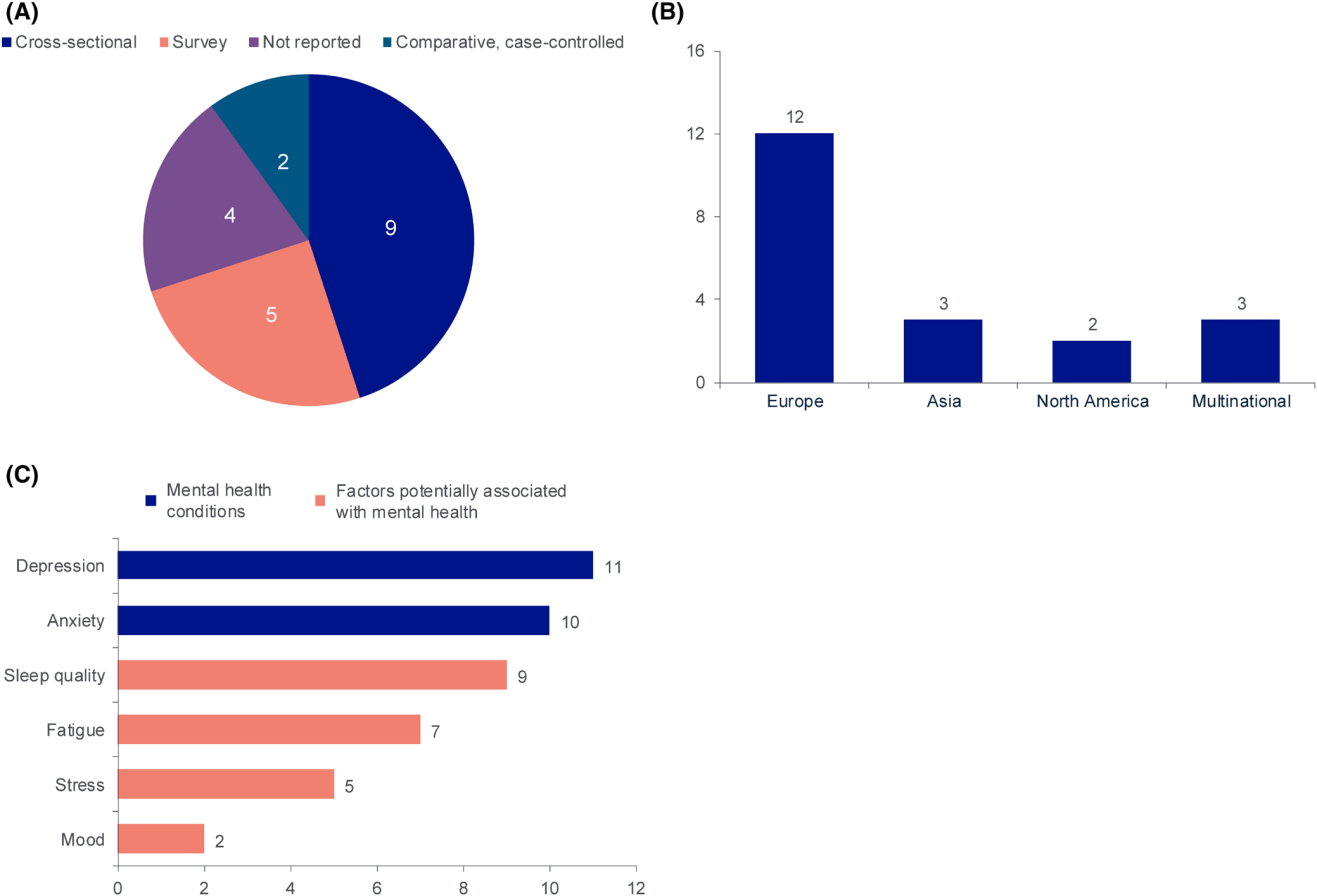
(A) Study design of included articles. (B) Geographic regions assessed in included articles. (C) Articles reporting mental health outcomes in caregivers of people with Dravet syndrome (*n* = 20). The Domaradzki and Walkowiak^14^, ^15^ published articles contain data from the same study. Some data in the Strzelczyk et al.^28^ published article are the same reported on in the Strzelczyk et al.^29^ published article.

The 20 included published articles were heterogenous in study design and included prospective cohorts, cross‐sectional analyses, surveys, comparative case–control studies, and qualitative methodologies such as semistructured interviews and focus groups (Table 2, Figure 2A). The majority of included articles were cross‐sectional studies^4^, ^16^, ^17^, ^18^, ^19^, ^20^, ^21^, ^29^, ^30^; surveys were also included.^12^, ^13^, ^14^, ^15^, ^26^ Three published articles reported data on associations between factors/variables related to mental health.^13^, ^16^, ^21^ Semistructured interviews^20^, ^22^, ^23^ and focus groups^24^ provided deeper insights into the lived experiences of caregivers. Comparative studies^25^, ^28^ offered additional context by contrasting findings from caregivers of people with DS with those from non‐DS populations (parents of minors without any diagnosed disease, caregivers of patients with drug‐resistant epilepsy, and caregivers of patients with epilepsy in seizure remission). The study design was not reported for four articles.^22^, ^23^, ^24^, ^27^

**TABLE 2:** Characteristics of the 20 included articles.

First author (year of publication) country	Year study was undertaken/data collected	Sample size, caregivers of people with DS	Study design	Characteristics of people with DS	Caregiver characteristics	Relevant assessment tools/instrument
Campbell (2018)^12^ USA	November 30, 2016 to December 20, 2016	30	Prospective survey	Age, years, mean (SD) [range]: 11.7 (5.8) [2–22]	NR	EQ‐5D‐5L
Cardenal‐Muñoz (2021)^13^ Spain	April 14, 2020 to May 17, 2020	69 families	Survey	Age, years, mean (SD) [median] (range): 12.6 (9.9) [10.1] (.6–49.3) Age, years, *n* (%): ≤2: 3 (4.3) 3–7: 18 (26.1) 8–17: 37 (50.7) ≥18: 13 (18.8) Sex, *n* (%): Male: 41 (59) Female: 28 (41)	NR	Online survey
Domaradzki (2023)^14^, ^a^, ^b^ Poland	December 30, 2022 to January 30, 2023	75	Survey	Age group, years, *n* (%): ≤1: 2 (2.5)^c^ 2–3: 3 (3.7) 4–5: 10 (12.5) 6–10: 28 (35) 11–18: 35 (43.8) Missing: 2 (2.5) Sex, *n* (%): Female: 37 (46.2) Male: 43 (53.8)	Age group, years, *n* (%): <30: 2 (2.7) 30–39: 32 (42.6) 40–49: 35 (46.7) ≥50: 6 (8.0) Caregiver, *n* (%) Mother: 66 (88.0) Father: 7 (9.3) Other relative [grandmother, sister]: 2 (2.7)	Self‐administered questionnaire
Domaradzki (2023)^15^, ^a^, ^b^ Poland	December 30, 2022 to February 28, 2023	75	Survey	Age, years, mean (SD) [range]: 9.7 (4.6) [1–18]^c^ Sex, *n* (%): Female: 37 (46.2) Male: 43 (53.8)	Age, years, mean (SD) [range]: 39.7 (6.5) [24–57] Sex, *n* (%): Female: 68 (90.7) Male: 7 (9.3)	Self‐administered questionnaire
Hesdorffer (2020)^16^ USA	NR	742: Aicardi syndrome 59; *CDKL5* mutation 18; Doose syndrome 42; **DS 100**; Dup15q mutation 36; hypothalamic hamartoma 40; West syndrome 38; Lennox–Gastaut syndrome 103; Ohtahara syndrome 7; *PCDH19* mutation 26; Phelan–McDermid syndrome 26; SCN8A 17; *SYNGAP* mutation 11; TSC 123; other encephalopathy or genetic mutation 59; other rare syndrome/diagnosis 37^d^	Cross‐sectional	Age, years, median (range): 8.6 (.3, 49.8) Age group, *n* (%): 0–23 months: 84 (11.3) 2–5 years: 182 (24.5) 6–9 years: 142 (19.1) 10–14 years: 152 (20.5) ≥15 years: 182 (24.5)	Age, years, median (range): 40 (22–79) Sex, *n* (%): Male: 60 (8.1) Female: 682 (91.9) Race, *n* (%): American Indian or Alaskan Native: 4 (.5) Asian: 15 (2.0) Black or African American: 9 (1.2) Native Hawaiian or Pacific Islander: 0 White or Caucasian: 675 (91.0) Mixed/other: 34 (4.6) Missing: 5 (.7) Ethnicity, *n* (%): Hispanic: 61 (8.2) Non‐Hispanic: 677 (91.2) Missing: 4 (.5) Relationship to affected person, *n* (%): Mother: 675 (91.0) Father: 60 (8.1) Grandmother: 7 (.9)	PROMIS Short Forms v1.0: Anxiety 8a Depression 8a Fatigue 8a Sleep Disturbance 8a Sleep‐Related Impairment 8a
Huang (2021)^17^ Taiwan	2019/2020	38	Cross‐sectional	Age, years, mean (SD) [range]: 10.5 (6.3) [1–28] Age group, years, *n* (%): 0–1: 1 (2.6) 2–5: 7 (18.4) 6–11: 18 (47.3) 12–17: 7 (18.4) ≥18: 5 (13.1) Sex, *n* (%): Female: 16 (42) Male: 21 (58)	NR	Questionnaire
Kalski (2019)^18^ Germany	April 2017 to January 2018; diaries were completed for 3 months after completion of the questionnaire	93	Prospective, cross‐sectional	Age, years, mean (SD) [median] (range): 10 (7.1) [8.5] (15 months–33.7 years) Sex, %: Male: 53 Female: 47	Age, years, mean (SD) [range]: Mothers: 42 (7.6) [28–62] Fathers: 45 (7.7) [29–70]	Questionnaire BDI‐II EQ‐5D‐3L
LoPresti (2024)^19^ Japan	September 2022 to November 2022	19	Cross‐sectional	Age, years, mean (SD) [median] (IQR): 8.7 (5.7) [8.0] (4.0–12.0) Sex, *n* (%): Female: 12 (63) Male: 7 (37)	Age, years, mean (SD) [median] (IQR): 42.3 (7.2) [42.0] (37.5–48.0) Sex, *n* (%): Female: 19 (100) Male: 0 Type of caregiver, *n* (%): Primary: 19 (100) Relationship with patient, *n* (%): Parent: 18 (95) Grandparent: 1 (5)	Questionnaire HADS
LoPresti (2024)^20^ Japan	April to November 2022	26: LGS 5; **DS 10**; TSC 11	Cross‐sectional	Age, years, mean (SD) [range]: 13.6 (10.0) [2–44] Age group, *n* (%): Pediatric (2–17 years): 19 (73) Adult (≥18 years): 7 (27) Sex, *n* (%): Male: 13 (50) Female: 13 (50)	Age, years, mean (SD) [range]: 45.9 (9.5) [29–68] Age group, *n* (%): Pediatric (2–17 years): 0 Adult (≥18 years): 26 (100) Sex, *n* (%): Male: 2 (8) Female: 24 (92)	Questionnaire Semistructured interview
Maltseva (2023)^21^ Germany	2019	108	Cross‐sectional	Age, years, mean (SD) [median] (range): 13.5 (10.0) [10.8] (1.2–46.2) Sex, *n* (%): Male: 53 (49.1) Female: 55 (50.9)	Age, years, mean (SD) [median]: Mothers: 44.7 (10.6) [42] Fathers: 47.3 (10.6) [53.5] Sex, *n* (%): Male: 8 (7.4) Female: 100 (92.6)	Retrospective questionnaire PSQI HADS BSFC
Nabbout (2018)^22^ France	NR	7	NR	Age group, years, *n* (%): 5–8: 3 (42.9) 9–11: 2 (28.6) 12–18: 2 (28.6) Sex, *n* (%): Male: 5 (71.4) Female: 2 (28.6)	Age, years, mean (range): 40.7 (35–53) Sex, *n* (%): Male: 4 (57.1) Female: 3 (42.8)	Semistructured interview
Nabbout (2019)^23^ multinational	NR	20	NR	Age group, years, *n* (%): 2–4: 5 (25) 5–8: 5 (25) 9–11: 6 (30) 12–18: 4 (20) Sex, *n* (%): Male: 8 (40) Female: 12 (60)	Age, years, mean (range): 43.4 (34–55) Sex, *n* (%): Male: 7 (35) Female: 13 (65) Country of residence, *n* (%): UK: 4 (20) USA: 4 (20) Australia: 8 (40) Italy: 4 (20)	Semistructured interview
Postma (2023)^24^ Belgium (Flanders) and the Netherlands	Focus groups conducted: June 29, 2021; July 15, 2021; and November 25, 2021	20	NR	Age, years, mean (SD) [median] (range): 11.8 (6.6) [10.5] (3–22) Age at diagnosis, years, mean (SD) [median] (range): 3.2 (3.9) [2.0] (.8–17) Age group, years, *n* (%): 3–13: 11 (55) 14–22: 9 (45)	Sex, *n* (%): Female parents: 20 (100) Male parents: 0	Semistructured focus groups
Salom (2023)^25^ Spain	NR	48	Comparative, case‐controlled	Age, years, mean (SD): 8.8 (4.8) Sex, *n*: Female: 25	Age, years, mean (SD): 41.7 (6.2) Sex, *n*: Female: 34	CRESIA
Skluzacek (2011)^26^ multinational	In 2009, parents of children with DS were invited to participate in surveys, which took place within a 2‐week period	86	Survey	NR	NR	Survey
Soto Jansson (2024)^27^ Sweden	October 15, 2018 to April 3, 2020	36 (for children with more than one caregiver, caregiver pairs or trios were labeled as one caregiver)	NR	Age at diagnosis, years, median (range): 2.20 (.53–10) Age at assessment, years, median (range): 8.09 (1.09–19) Sex, *n* (%): Female: 16 (44) Male: 20 (56)	Age, years, mean (range): Mothers, *n* = 30: 39.04 (27–54) Fathers, *n* = 13: 42.97 (29–58) Parent born in Sweden, *n* (%): Mother, *n* = 30: 26 (87) Father, *n* = 13: 11 (85)	Investigator developed interview questions on impact of DS on the family
Strzelczyk (2019)^28^, ^e^ Germany	Three studies: The EpiPaed study was completed in 2011 The study in adults was completed in 2013 The German DS study was completed in 2018	93 (93 patients with DS and their caregivers were matched by age and gender with responses from 93 patients with drug‐resistant epilepsy and 93 patients in seizure remission collected in three independent studies)	Comparative, case‐controlled	Age, years, mean [median]: Pediatric cohort (*n* = 82): 8.1 [7.4] Adult cohort (*n* = 11): 24.6 [23.3] Sex (%): Pediatric female: 45 Adult female: 64	NR	Questionnaire BDI‐II EQ‐5D‐3L
Strzelczyk (2019)^29^ Germany	Questionnaires were completed between April 2017 and January 2018; diaries were kept by caregivers for 3 months after the period covered by the questionnaire (latest April 2018)	93	Cross‐sectional	Age, years, mean (SD) [median] (range): 10.1 (7.1) [8.7] (15 months–33.7 years) Sex, *n* (%): Male: 49 (53)	Age, years, mean (SD) [median]: Mother, *n* = 93: 42.1 (7.6) [41.5] Father, *n* = 93: 45.2 (7.7) [45.0]	Questionnaire BDI‐II EQ‐5D‐3L
Villas (2017)^4^ multinational	March to May 2016	256	Cross‐sectional	Age group, years, *n* (%): <1: 1 (.4) 1–3: 39 (15) 4–6: 49 (19) 7–10: 72 (28) 11–15: 37 (14) 16–25: 54 (21) ≥26: 4 (2) Sex, *n* (%): Female: 140 (55)	Country of residence, *n* (%): USA: 180 (70) UK: 32 (13) Europe: 13 (5) Australia: 12 (5) Canada: 9 (4) Central/South America: 4 (2) Other: 6 (2) Relationship to patient: birth parent, *n* (%): 239 (93) Parent is *SCN1A*+, *n* (%): 16 (6)	Online survey
Gil‐Nagel (2023)^30^ Spain	October 2020 to March 2021	80	Cross‐sectional	Age, years, mean (SD) [median] (IQR): 12.7 (9.6) [10.8] (6.5–14.9) Sex, *n* (%): Female: 38 (47.5)	NR	CarerQoL

Abbreviations: BDI‐II, Beck Depression Inventory‐II; BSFC, Burden Scale for Family Caregivers; CarerQoL, Care‐Related Quality of Life; *CDKL5*, cyclin dependent kinase like 5; CRESIA, Childhood Rare Epilepsy Social Impact Assessment; DS, Dravet syndrome; Dup15q, chromosome 15q11.2–13.1 duplication syndrome; EQ‐5D‐3L, European Quality of Life 5 Dimensions 3 Level Version; EQ‐5D‐5L, European Quality of Life 5 Dimensions 5 Level Version; HADS, Hospital Anxiety and Depression Scale; IQR, interquartile range; LGS, Lennox–Gastaut syndrome; NR, not reported; *PCDH19*, protocadherin‐19; PROMIS, Patient‐Reported Outcomes Measurement Information System; PSQI, Pittsburg Sleep Quality Index; *SCN1A*, sodium voltage‐gated channel alpha subunit 1; *SCN8A*, sodium channel protein type 8 subunit alpha; *SYNGAP*, synaptic Ras GTPase‐activating protein 1; TSC, tuberous sclerosis complex.

^a^ Domaradski and Walkowiak^14^, ^15^ published articles contain data from the same study.

^b^ Some data/information in the Domaradski and Walkowiak^14^, ^15^ published articles have been updated since the original publication, as confirmed by the corresponding author.

^c^ The data presented in this table are as reported in the Domaradski and Walkowiak^14^, ^15^ published articles (of note that in the Domaradski and Walkowiak^14^ article there were two patients [2.5%] reported to be in the ≤1 year of age group; whereas in the Domaradski and Walkowiak^15^ article, the age range of children was reported to be 1–18 years).

^d^ Angelman syndrome (*n* = 2), congenital bilateral perisylvian syndrome (*n* = 2), electrical status epilepticus in slow wave sleep (*n* = 5), Jeavons syndrome (*n* = 3), KCNQ2 (*n* = 2), Landau Kleffner syndrome (*n* = 4), lissencephaly (*n* = 1), progressive or other myoclonic epilepsy (*n* = 4), Rasmussen encephalopathy (*n* = 2), Ring chromosome 14 syndrome (*n* = 3), Ring chromosome 20 syndrome (*n* = 2), SCN2A (*n* = 3), SLC13A5 (*n* = 2), Unverricht Lundborg disease (*n* = 2).

^e^ The Strzelczyk et al.^28^ published article contains data from a study that was also published in the Strzelczyk et al.^29^ article.

Geographic regions included North America, with two studies conducted in the United States,^12^, ^16^ and Europe, with three studies (four articles) from Germany,^18^, ^21^, ^28^, ^29^ three studies from Spain,^13^, ^25^, ^30^ and one study (two articles) from Poland^14^, ^15^ (Figure 2B). One study each was identified from Belgium (Flanders) and the Netherlands,^24^ Sweden,^27^ and France.^22^ Other regions represented included Asia, with studies from Taiwan^17^ and Japan.^19^, ^20^ Finally, there were three multinational studies.^4^, ^23^, ^26^

Study sample sizes varied widely, ranging from as few as seven^22^ to as many as 256^4^ caregivers of people with DS. A total of six articles did not report caregiver characteristics.^12^, ^13^, ^17^, ^18^, ^26^, ^30^ Most caregivers were female,^14^, ^15^, ^16^, ^19^, ^20^, ^21^, ^23^, ^24^, ^25^, ^27^ comprising between 42.8%^22^ and 100%^19^ of caregivers per study. A high percentage of caregivers were mothers/female parents (up to 88%^14^ and 95%^19^). Between 0%^19^, ^24^ and 57.1%^22^ of caregivers were male. Fathers/male parents were reported as caregivers in six studies^14^, ^16^, ^18^, ^21^, ^27^, ^29^; they typically constituted a smaller proportion of caregivers, ranging from 0%^19^, ^24^ to 50% per study.^29^ Other caregivers included relatives such as grandmothers, siblings, and other family members.^14^, ^16^, ^19^

Validated instruments used in different studies included the Beck Depression Inventory‐II (BDI‐II),^18^, ^28^, ^29^ which measures symptoms of depression, and the Hospital Anxiety and Depression Scale (HADS),^19^, ^21^ which assesses anxiety and depression (Table 3). Although the European Quality of Life 5 Dimensions (EQ‐5D) questionnaire was the most commonly used instrument to assess HRQoL in caregivers,^12^, ^18^, ^28^, ^29^ only two articles reported on the anxiety/depression domain of the EQ‐5D.^12^, ^29^ Additionally, Patient‐Reported Outcomes Measurement Information System (PROMIS) Short Forms were used to assess anxiety, depression, fatigue, and sleep disturbances,^16^ and in another study the Pittsburgh Sleep Quality Index (PSQI) was used to assess sleep disturbances in caregivers.^21^ More than half of studies did not use a validated instrument to assess mental health in caregivers. Nine studies used questionnaires,^14^, ^15^, ^17^, ^18^, ^19^, ^20^, ^21^, ^29^ and three studies each used surveys^4^, ^13^, ^26^ or semistructured interviews^20^, ^22^, ^23^ to assess the mental health of caregivers.

**TABLE 3:** Mental health outcomes in caregivers of people with DS.

First author (year) country	Sample size, caregivers of people with DS	Mental health conditions		Factors potentially associated with mental health					Key findings	
		Depression	Anxiety	Fatigue	Sleep quality	Stress	Mood	Others		
Campbell (2018)^12^ USA	30	Yes	Yes	No	No	No	No		EQ‐5D domain of anxiety/depression: Not anxious/depressed: 30% At least slightly anxious/depressed: 70% At least moderately anxious/depressed: 33% EQoL Index, mean (SD): .78 (.17)	
Cardenal‐Muñoz (2021)^13^ Spain	69 families	No	Yes	No	No	No	Yes		76.1% of caregivers reported changes in their mood; 80% reported new symptoms of anxiety (both deemed unrelated to type of lockdown or seizures experienced during the COVID‑19 lockdown) Observed relationship between the caregiver's mood (but not anxiety) and the patient's behavior (*p* = .012) Observed relationship between caregiver's mood with nocturnal seizures prior to lockdown (3 months; *p* = .037)	
Domaradzki (2023)^14^ Poland	75	No	No	Yes	Yes	No	No	Deterioration of mental health	Family caregivers reported often or always experiencing fatigue (84%) and a deterioration of mental health (60%) when asked about their problems 42.7% of caregivers answered often or always regarding experiencing problems with sleeping/insomnia	
Domaradzki (2023)^15^ Poland	75	Yes	Yes	Yes	Yes	Yes	No	Mental exhaustion Solitude and isolation Hopelessness Helplessness Nervousness/impulsivity	Percentage of caregivers who answered often or always regarding feelings resulting from caregiving: Physical fatigue: 81.3% Mental exhaustion: 80% Stress: 38.7% Not coping with stress: 46.7% Solitude and isolation: 41.3% Sadness and depression: 50.7% Hopelessness and a loss of meaning: 38.7% Percentage of caregivers who answered often or always regarding emotional states experienced by caregivers: Helplessness: 72% Anxiety and fear: 68% Sadness/depression: 52% Nervousness/impulsivity: 50.7% Loneliness: 46.7% Hopelessness: 45.3% 46.7% of caregivers answered rather bad or very bad regarding self‐reported quality of sleep Caregiver self‐rated quality of sleep, *n* (%): Very bad: 9 (12) Rather bad: 26 (34.7) Neither good nor bad/don't know: 7 (9.3) Rather good: 29 (38.7) Very good: 4 (5.3)	
Hesdorffer (2020)^16^ USA	742 (DS 100)	Yes	Yes	Yes	Yes	No	No	Companionship	**FATIGUE** **Caregiver fatigue PROMIS *T*‐scores, ≤ 60 (no); > 60 (yes), *n* (%)** **Characteristics of the child:** ⚬ Child has difficulty falling asleep: Fatigue *T*‐score: ≤ 60: 156 (39.5); > 60: 141 (47) ⚬ Child excess daytime sleepiness: Fatigue *T*‐score: ≤ 60: 109 (28); > 60: 112 (37.8) ⚬ Child frequent nighttime awakenings: Fatigue *T*‐score: ≤ 60: 173 (43.8); > 60: 165 (55.2) ⚬ Child very restless sleep: Fatigue *T*‐score: ≤ 60: 121 (31.1); > 60: 138 (47.3) ⚬ Child any sleep condition listed above: Fatigue *T*‐score: ≤ 60: 221 (55.4); > 60: 202 (67.1) **Methods for nighttime monitoring:** ⚬ Someone shares a room or bed with the person with epilepsy: Fatigue *T*‐score: ≤ 60: 91 (22.4); > 60: 87 (28.2) ⚬ Audio monitor or seizure alert device or leave door open: Fatigue *T*‐score: ≤ 60: 231 (56.9); > 60: 174 (56.3) ⚬ Watch or check frequently: Fatigue *T*‐score: ≤ 60: 5 (1.2); > 60: 12 (4.2) **Caregiver PROMIS *T*‐scores:** ⚬ Depression *T*‐score > 60: Fatigue *T* score: ≤ 60: 34 (8.2); > 60: 101 (32.5) ⚬ Anxiety *T*‐score > 60: Fatigue *T*‐score: ≤ 60: 75 (18.2); > 60: 176 (56.4) ⚬ Cognition *T*‐score > 60: Fatigue *T*‐score: ≤ 60: 146 (35.4); > 60: 20 (6.4) ⚬ Companionship *T*‐score > 60: Fatigue *T*‐score: ≤ 60: 133 (32.3); > 60: 44 (14.1)	**Significant risk factors for caregiver fatigue (logistic regression for risk factors by fatigue *T*‐score [≤ 60 (no); > 60 (yes)])** ***T*‐score ≤ 60 vs. > 60, aOR (95% CI), *p*‐value** **Characteristics of the child:** ⚬ Child has difficulty falling asleep: 1.5 (1.1–2.07), *p* = .01 ⚬ Child excess daytime sleepiness: 1.8 (1.3–2.5), *p* = .001 ⚬ Child frequent nighttime awakenings: 1.8 (1.3–2.5), *p* < .001 ⚬ Child very restless sleep: 2.2 (1.6–3.1), *p* < .001 ⚬ Child any sleep condition listed above: 2.0 (1.4–2.7), *p* < .001 **Methods for nighttime monitoring:** ⚬ Someone shares a room or bed with the person with epilepsy: 1.9 (1.2–3.3), *p* = .01 ⚬ Audio monitor or seizure alert device or leave door open: 1.7 (1.1–2.7), *p* = .03 ⚬ Watch or check frequently: 5.3 (1.8–17.9), *p* = .04 **Caregiver PROMIS *T*‐scores:** ⚬ Depression *T*‐score > 60: 6.0 (3.9–9.6), *p* < .001 ⚬ Anxiety *T*‐score > 60: 6.0 (4.2–8.7), *p* < .001 **Significant protective factors for fatigue** **Caregiver PROMIS *T*‐scores:** ⚬ Cognition *T*‐score > 60: .1 (.1–.2), *p* < .001 ⚬ Companionship *T*‐score > 60: .3 (.2–.4), *p* < .001
									**SLEEP DISTURBANCE** **Caregiver sleep disturbance PROMIS *T*‐scores, ≤ 60 (no); > 60 (yes), *n* (%)** **Characteristics of the child:** ⚬ Child has any of the following: difficulty falling asleep, excess daytime sleepiness, frequent nighttime awakenings, and very restless sleep: Sleep disturbance *T*‐score: ≤ 60: 288 (56.4); > 60: 137 (70.6) **Methods for nighttime monitoring:** ⚬ Someone shares a room or bed with the person with epilepsy: Sleep disturbance *T*‐score: ≤ 60: 126 (24.1); > 60: 54 (27.1) ⚬ Audio monitor or seizure alert device or leave door open: Sleep disturbance *T*‐score: ≤ 60: 282 (54.0); > 60: 126 (63.3) **Caregiver PROMIS *T*‐scores:** ⚬ Depression *T*‐score > 60: Sleep disturbance *T*‐score: ≤ 60: 75 (14.2); > 60: 60 (29.7) ⚬ Anxiety *T*‐score > 60: Sleep disturbance *T*‐score: ≤ 60: 142 (27.9); > 60: 110 (54.5) ⚬ Cognition *T*‐score > 60: Sleep disturbance *T*‐score: ≤ 60: 150 (28.4); > 60: 17 (8.4) ⚬ Companionship *T*‐score > 60: Sleep disturbance *T*‐score: ≤ 60: 151 (28.6); > 60: 26 (12.9)	**Significant risk factors for caregiver sleep disturbance (logistic regression for risk factors by sleep disturbance *T*‐score [≤ 60 (no); > 60 (yes)])** ***T*‐score ≤ 60 vs. > 60, aOR (95% CI), *p*‐value** **Characteristics of the child:** ⚬ Child has any of the following: difficulty falling asleep, excess daytime sleepiness, frequent nighttime awakenings, and very restless sleep: 1.9 (1.3–2.8), *p* < .001 **Methods for nighttime monitoring:** ⚬ Someone shares a room or bed with the person with epilepsy: 2.8 (1.5–5.6), *p* = .002 ⚬ Audio monitor or seizure alert device or leave door open: 3.1 (1.7–5.8), *p* < .001 **Caregiver PROMIS *T‐*scores:** ⚬ Depression *T‐*score > 60: 2.8 (1.9–4.2), *p* < .001 ⚬ Anxiety *T*‐score > 60: 3.6 (2.5–5.1), *p* < .001 **Significant protective factors for sleep disturbance** **Caregiver PROMIS *T*‐scores:** ⚬ Cognition *T*‐score > 60: .2 (.1–.4), *p* < .001 ⚬ Companionship *T*‐score > 60: .4 (.2–.6), *p* < .001
									**SLEEP IMPAIRMENT** **Caregiver sleep impairment PROMIS *T*‐scores, ≤ 60 (no); > 60 (yes), *n* (%)** **Characteristics of the child:** ⚬ Child has any of the following: difficulty falling asleep, excess daytime sleepiness, frequent nighttime awakenings, and very restless sleep: Sleep impairment *T*‐score: ≤ 60: 261 (56.9); > 60: 164 (66.7) **Caregiver PROMIS *T*‐scores:** ⚬ Depression *T*‐score > 60: Sleep impairment *T*‐score: ≤ 60: 50 (10.5); > 60: 85 (33.5) ⚬ Anxiety *T*‐score > 60: Sleep impairment *T*‐score: ≤ 60: 108 (22.7); > 60: 144 (56.5) ⚬ Cognition *T*‐score > 60: Sleep impairment *T*‐score: ≤ 60: 154 (32.4); > 60: 13 (5.1) ⚬ Companionship *T*‐score > 60: Sleep impairment *T*‐score: ≤ 60: 144 (30.3); > 60: 33 (12.9)	**Significant risk factors for caregiver sleep impairment (logistic regression for risk factors by sleep impairment *T*‐score [≤ 60 (no); > 60 (yes)])** ***T*‐score ≤ 60 vs. > 60, aOR (95% CI), *p*‐value** **Characteristics of the child:** ⚬ Child has any of the following: difficulty falling asleep, excess daytime sleepiness, frequent nighttime awakenings, and very restless sleep: 1.7 (1.2–2.4), *p* = .003 **Caregiver PROMIS *T*‐scores:** ⚬ Depression *T*‐score > 60: 4.5 (3.0–6.7), *p* < .001 ⚬ Anxiety *T*‐score > 60: 4.3 (3.1–6.1), *p* < .001 **Significant protective factors for sleep impairment** **Caregiver PROMIS *T*‐scores:** ⚬ Cognition *T*‐score > 60: .1 (.1–.2), *p* < .001 ⚬ Companionship *T‐*score > 60: .3 (.2–.4), *p* < .001
Huang (2021)^17^ Taiwan	38	Yes	Yes	No	No	No	No	Isolation	Having suffered from depressed mood was reported by almost half of the caregivers (47%) After seizure control, anxiety/depression/isolation was among the most common caregiver concerns for 5.2% (*n* = 2/38) of caregivers	
Kalski (2019)^18^ Germany	93	Yes	No	No	No	No	No		EQ‐5D‐3L score, mean (SD) [range]: .9 (.18) [.3–1] (similar to the adult German population) BDI‐II score, mean (SD) [range]: 15.4 (9.7) [0–43]; 46% of parents exceeded the cutoff score of ≥14 ⚬ BDI‐II, *n* (%): Mild symptoms: 20 (22) Moderate symptoms: 13 (15) Severe symptoms: 8 (9)	
LoPresti (2024)^19^ Japan	19	Yes	Yes	No	Yes	Yes	No		Symptoms experienced by caregivers, *n* (%): Stress: 10 (53) Sleep problems: 8 (42) Anxiety: 7 (37) Depression: 1 (5) HADS score (*n* = 19), mean (SD) [median] (IQR) Total score: 20.0 (9.0) [20.0] (14.0–25.5) Depression: 8.3 (4.7) [8.0] (5.5–10.0) Anxiety: 11.6 (4.7) [12.0] (8.0–15.0) PedsQL FIM score (*n* = 18), mean (SD) [median] (IQR) Parent HRQoL summary: 54.2 (18.9) [54.4] (46.9–60.9) Family functioning summary: 58.9 (19.9) [62.5] (56.3–71.1) Total: 53.0 (16.4) [52.4] (46.0–62.9)	
LoPresti (2024)^20^ Japan	26: LGS 5; **DS 10**; TSC 11	Yes	Yes	No	Yes	No	No	Lowest emotional state	Around the time of onset/diagnosis, one caregiver described isolation, stating: “It was very hard to go out where there were other people and, little by little, I became introverted. I interacted with people less. This was when I had to constantly be watchful [of the patient]. I was at home all the time and couldn't work. I think I was depressed” At the time of diagnosis caregivers reported anxiety as the prevailing emotion At time of diagnosis/onset: “Caregivers also described a sense of anxiety about the future as they learned more about the illness” Postdiagnosis: “Several caregivers related that their lowest emotional state coincided with periods when they had spent a lot of time caring for the patient. Many felt depressed when they had to resign from their jobs” Current emotional burden: “Many caregivers stated that, similar to the period after diagnosis, caregiving is a burden when seizures are uncontrolled, reporting irregular sleep or sleep deprivation due to nighttime seizures”	
Maltseva (2023)^21^ Germany	108	Yes	Yes	No	Yes	No	No		PSQI score: ⚬ Overall score, mean (SD) [median] (range): 8.7 (3.5) [8.5] (2–16) ⚬ PSQI score range, *n* (%): 0−5: 25 (23.1) 6−10 (abnormal sleep quality): 50 (46.3) 11−21 (severe sleep disorder): 33 (30.6) ⚬ Univariate analyses: Caregivers with higher PSQI scores were significantly more likely to report at least moderate sleep disturbances (*p* = .02) • HADS scores: ⚬ HADS anxiety score, mean (SD) [median] (range): 9.3 (4.3) [9] (1–20) ⚬ HADS anxiety score range, *n* (%): 0−7 (normal): 39 (38.2) 8−10 (borderline): 27 (26.5) 11−21 (abnormal): 36 (35.3) ⚬ Univariate analyses: Caregivers with higher HADS anxiety scores were significantly more likely to report at least moderate sleep disturbances (*p* = .03) Caregivers with higher HADS anxiety scores were significantly more likely to report the patient slept in the same bedroom as them (*p* = .03) ⚬ HADS depression score, mean (SD) [median] (range): 7.9 (3.7) [7] (1–20) ⚬ HADS depression score range, *n* (%): 0−7 (normal): 53 (49.1) 8−10 (borderline): 30 (27.8) 11−21 (abnormal): 25 (23.1) ⚬ Univariate analyses: Caregivers with higher HADS depression scores were significantly more likely to report the patient slept in the same bedroom as them (*p* = .01) Caregivers with higher HADS depression scores were significantly more likely to report a higher frequency of patient GTCS (daily or weekly; *p* = .01) • BSFC score: ⚬ Overall score, mean (SD) [median] (range): 41.7 (11.7) [40] (17–64) ⚬ Score range, *n* (%): 0−41 (normal): 59 (54.6) 42−55 (moderate): 36 (33.3) 56−84 (severe to very severe): 13 (12.0) ⚬ Univariate analyses: Caregivers with higher BSFC scores were significantly more likely to report at least moderate sleep disturbances (*p* = .02) Caregivers with higher BSFC scores were significantly more likely to report a higher frequency of patient nocturnal seizures (daily or weekly; *p* = .03) and a higher frequency of patient GTCS (daily or weekly; *p* = .02) • Higher PSQI scores correlated significantly with higher BSFC score (*r* = .358, *p* < .001), HADS anxiety scores (*r* = .456, *p* < .001), and HADS depression scores (*r* = .215, *p* = .026)	
Nabbout (2018)^22^ France	7	No	No	Yes	Yes	No	No	Worry Uncertainty	Impacts on caregivers involved in the care of children with DS, *n* (reported by ≥4 caregivers and health care professionals): Fear/afraid: 8 (7 caregivers; 1 health care professional) Worry: 7 (7 caregivers) Tiredness: 7 (5 caregivers; 2 health care professionals) Sleep disturbed: 5 (5 caregivers) Annoyed/frustrated: 4 (4 caregivers) Uncertainty: 4 (4 caregivers)	
Nabbout (2019)^23^ multinational	20	No	No	Yes	Yes	Yes	No	Worry	Reported impacts by caregivers, *n* (%): Sleep disturbance: 15 (75) Emotional experience: 10 (50) Between‐country differences for caregiver impacts (number of caregivers reporting impacts by country): Worry: Australia, *n* = 6; UK, *n* = 1; USA, *n* = 1; Italy, *n* = 1 Stress: Australia, *n* = 3; UK, *n* = 0; USA, *n* = 2; Italy, *n* = 0 Feeling tired (fatigue): Australia, *n* = 2; UK, *n* = 1; USA, *n* = 1; Italy, *n* = 3	
Postma (2023)^24^ Belgium (Flanders) and the Netherlands	20	No	No	Yes	No	Yes	No		Feeling exhausted was reported by many parents; one caregiver described exhaustion: “The first two years we were just surviving. We were completely exhausted to be honest” Parents discussed ways of parenting a child with DS influenced their own mental health; they reported refractory epilepsy and life‐threatening situations were very stressful	
Salom (2023)^25^ Spain	48	No	No	No	No	No	No	Emotional state	CRESIA psychological scale/domain, mean (SD) [median]: 2.91 (.49) [2.92] ⚬ Emotional state subscale, mean (SD) [median]: 2.90 (.51) [2.92] ⚬ Self‐concept subscale, mean (SD) [median]: 2.94 (.57) [2.95] Significant impact in the CRESIA psychological scale/domain, affecting caregivers' emotional state: CRESIA emotional state subscale differed between caregivers of children with DS vs. control group (caregivers of minors without any diagnosed disease); *t*(94) = 4.59, *p* < .001	
Skluzacek (2011)^26^ multinational	86	No	No	No	No	Yes	No	Grief	89% of parents affirmed the need to have support for the management of ongoing stress associated with caring for their child 57 parents responded to questions regarding grief and adaptation with respect to their children's DS: ⚬ 86% acknowledged experiencing grief regarding their child's condition	
Soto Jansson (2024)^27^ Sweden	36	No	No	Yes	Yes	No	Yes	General mental health	Themes related to fatigue and sleep: ⚬ The requirement for constant monitoring because of the child's behavioral difficulties lead to parental exhaustion ⚬ Requirement of the parent to sleep with the child ⚬ Parents affected by frequent night waking Themes related to mental health: ⚬ Parents have poorer physical and mental health, with impacts on energy and mood ⚬ Impacts on family's social life, finances, parental sleep, and relationships	
Strzelczyk (2019)^28^ Germany	93	Yes	No	No	No	No	No		Depression (BDI‐II scores) symptom severity, *n* (%): No depressive symptoms (0−13): 37 (50) Mild depressive symptoms (14−19): 19 (26) Moderate depressive symptoms (20−28): 12 (16) Severe depressive symptoms (29−63): 6 (8) Caregivers with BDI‐II score of 0 (no depressive symptoms), *n* (%): 1 (1.0) Mean overall BDI‐II score: 14.9 Mean BDI‐II score was significantly higher in caregivers of children with DS (14.9) than in caregivers of children with DRE (9.4; *p* < .001) or SR (6.9: *p* < .001) A higher percentage of DS caregivers had moderate to severe depression symptoms (24%) compared with caregivers of children with DRE (11%) or SR (5%) Mean [SD] carer EQ‐5D‐3L score was significantly lower in caregivers of children with DS (.90 [.18]) than caregivers of children with SR (.96 [.07], *p* < .01), but similar to caregivers of children with DRE (.94 [.10]) Mean carer EQ‐VAS score was significantly lower in caregivers of children with DS (73) than caregivers of children with SR (80, *p* < .01), but similar to caregivers of children with DRE (76)	
Strzelczyk (2019)^29^ Germany	93	Yes	Yes	No	No	No	No		Depression (BDI‐II scores) symptom severity, *n* (%): No depression (0−13): 41 (44) Mild depression (14−19): 20 (22) Moderate depression (20−28): 14 (15) Severe depression (29−63): 8 (9) No response: 10 (11) Caregiver EQ‐5D: EQ‐5D‐3L, mean (SD) [median] (range): .9 (.18) [.9] (.3–1) EQ‐VAS, mean (SD) [median] (range): 71.3 (18) [73.0] (19–100) Mean German population norm EQ‐5D: EQ‐5D‐3L: .9 EQ‐VAS: 77.3 Higher levels of problems in anxiety/depression component of the EQ‐5D‐3L were reported by caregivers of people with DS compared with German population norms (38.2% vs. 4.3%)	
Villas (2017)^4^ multinational	256	Yes	Yes	No	No	No	No	Concerns about the emotional impact on siblings	After seizure control, anxiety/depression/isolation was one of the most common concerns for 19 caregivers 66% (*n* = 102/155) of caregivers reported having experienced depression; however, only 26% (*n* = 41/155) had received any form of family therapy 74% (*n* = 114/154) of caregivers reported having/having had concerns about the emotional impact on siblings	
Gil‐Nagel (2023)^30^ Spain	80	No	No	No	No	No	No	General mental health Fulfillment	CarerQoL‐7D, *n* (%): Fulfillment: No, 8 (10.0); Some, 18 (22.5); A lot, 54 (67.5) Mental health problems: No, 25 (31.3); Some, 37 (46.3); A lot, 18 (22.5) CarerQoL‐VAS score, mean (SD): 7.2 (2.1)	

Abbreviations: aOR, adjusted odds ratio; BDI‐II, Beck Depression Inventory‐II; BSFC, Burden Scale for Family Caregivers; CarerQoL‐7D, Care‐Related Quality of Life 7 Dimensions; CarerQoL‐VAS, Care‐Related Quality of Life Visual Analogue Scale; CI, confidence interval; COVID‐19, coronavirus disease 2019; CRESIA, Childhood Rare Epilepsy Social Impact Assessment; DRE, drug‐resistant epilepsy; DS, Dravet syndrome; EQ‐5D, European Quality of Life 5 Dimensions; EQ‐5D‐3L, European Quality of Life 5 Dimensions 3 Level Version; EQoL, EQ‐5D summary index; EQ‐VAS, European Quality of Life Visual Analogue Scale; GTCS, generalized tonic–clonic seizures; HADS, Hospital Anxiety and Depression Scale; HRQoL, Health‐Related Quality of Life; IQR, interquartile range; LGS, Lennox–Gastaut syndrome; PedsQL FIM, Pediatric Quality of Life Inventory Family Impact Module; PROMIS, Patient‐Reported Outcomes Measurement Information System; PSQI, Pittsburg Sleep Quality Index; SR, seizure remission; TCS, tuberous sclerosis complex.

### 3.3 Mental health outcomes assessed for caregivers of people with DS

#### 3.3.1 Mental health conditions: Depression and anxiety

Depression and anxiety, which are categorized as mental health conditions, were the most commonly reported mental health outcomes, reported in 11^4^, ^12^, ^15^, ^16^, ^17^, ^18^, ^19^, ^20^, ^21^, ^28^, ^29^ and 10 articles,^4^, ^12^, ^13^, ^15^, ^16^, ^17^, ^19^, ^20^, ^21^, ^29^ respectively (Figure 2C, Table 3). The prevalence of depression and anxiety among caregivers varied widely across articles, ranging from 5%^19^ to 66%,^4^ and 5.2%^17^ to 80%,^13^ respectively.

Three articles reported on the use of BDI‐II to assess depressive symptoms among caregivers.^18^, ^28^, ^29^ A study undertaken in Germany reported a mean overall BDI‐II score of 15.4 (indicating mild depressive symptoms) for caregivers.^18^ When BDI‐II scores of caregivers were stratified by the age of people with DS receiving care, there was a higher percentage of BDI‐II scores ≥ 20 (indicating moderate or severe depression) for those caregivers of people with DS who were 12–17 and ≥18 years of age than those who were 6–11 and ≤5 years of age.^18^ Another German study also reported similar results, with a mean overall BDI‐II score of 14.9 (indicating mild depressive symptoms) for caregivers.^28^ When compared with cohorts of caregivers of age‐ and sex‐matched patients with drug‐resistant epilepsy (mean overall BDI‐II score = 9.4), and with caregivers of age‐ and sex‐matched patients with epilepsy in seizure remission (mean overall BDI‐II score = 6.9), caregivers of people with DS had significantly higher levels of depression (*p* < .001).^28^

Two articles reported on the use of HADS to assess caregiver emotional well‐being and symptoms of depression.^19^, ^21^ One study on caregivers residing in Japan reported a median HADS depression score of 8.0 (indicating borderline abnormal levels of depression) and a median HADS anxiety score of 12.0 (indicating abnormal levels of anxiety) for caregivers of people with rare epilepsy syndromes including DS.^19^ An increased number of caregiving hours (≥21 vs. <21 h in the previous week) was associated with increased caregiver levels of depression and anxiety for caregivers, as shown by increases in HADS depression (median [IQR] = 8.0 [6.0–10.0] vs. 3.5 [3.0–7.3], *p* = .0282) and HADS anxiety scores (12.0 [8.0–15.0] vs. 4.5 [3.3–7.5], *p* = .0039).^19^ A study on caregivers of individuals with DS in Germany reported a median HADS depression score of 7 (indicating normal levels of depression) and a median HADS anxiety score of 9 (indicating borderline abnormal levels of anxiety).^21^ Increased levels of depression were reported relative to the general population.^21^ When examining HADS depression and HADS anxiety scores, 50.9% and 61.8% of caregivers scored above 8 (score of ≥8 included those with borderline and abnormal levels of depression or anxiety) for depression and anxiety, respectively, which the authors deemed to be a tendency toward severe depression and anxiety symptoms.^21^ The median HADS depression scores from these studies indicated suspected depression and confirmed anxiety among these caregivers.

A study from the United States reported the EQ‐5D domains of anxiety/depression had the greatest impact on caregivers (70% of respondents were at least slightly anxious/depressed, and 33% were at least moderately anxious/depressed).^12^ Furthermore, a prospective German study reported higher levels of anxiety/depression in the anxiety/depression component of the EQ‐5D 3 Level Version for caregivers of people with DS compared with German population norms.^29^

#### 3.3.2 Factors potentially associated with mental health

Commonly reported outcomes categorized as factors potentially associated with mental health included sleep quality affecting mental health,^14^, ^15^, ^16^, ^19^, ^20^, ^21^, ^22^, ^23^, ^27^ fatigue,^14^, ^15^, ^16^, ^22^, ^23^, ^24^, ^27^ and stress^15^, ^19^, ^23^, ^24^, ^26^ (Figure 2C, Table 3).

##### Sleep quality

Many caregivers reported poor sleep quality, often linked to nighttime monitoring and frequent night waking of people with DS, with issues such as sleep disturbances, sleep impairment, or insomnia due to caregiving responsibilities.^14^, ^16^, ^21^, ^22^, ^23^, ^27^ In one article from a Polish study, 42.7% of caregivers answered that they often or always experienced problems with sleeping/insomnia.^14^ Similarly, in a separate article on the same Polish study, almost half of caregivers self‐reported their quality of sleep as rather bad or very bad.^15^ Sleep disturbance was reported by 75% (*n* = 15/20) of caregivers in one multinational study.^23^ Two studies reported on how other mental health outcomes including depression and anxiety were related to poor sleep quality.^16^, ^21^ A large, cross‐sectional US study of caregivers of people with rare epilepsy syndromes (including DS) hypothesized that caregiver sleep would be negatively affected by nocturnal monitoring strategies and perceived mental burden (related to seizure worry, SUDEP, and nocturnal seizure burden).^16^ Results showed several risk factors, including caregiver PROMIS depression *T* scores > 60 and anxiety *T* scores > 60, were significantly associated with caregiver sleep disturbance and sleep impairment.^16^ However, caregiver PROMIS cognition *T* scores > 60 and PROMIS companionship *T* scores > 60 were found to be significant protective factors for caregiver sleep disturbance and sleep impairment.^16^

A cross‐sectional German study reported 76.9% of caregivers had a PSQI score of 6 or above, indicating abnormal sleep quality, and 30.6% of caregivers had a PSQI score of 11 or above, indicating severe sleep disorder.^21^

The overall mean (SD) PSQI score was 8.7 (3.5) for caregivers of people with DS compared with a mean score of 5 (3.4) among the general German population, and higher PSQI scores among caregivers were significantly correlated with caregivers reporting at least moderate sleep disturbances.^21^ Caregivers with higher Burden Scale for Family Caregivers scores were significantly more likely to report at least moderate sleep disturbances.^21^ Higher HADS depression and anxiety scores were observed among caregivers who slept in the same bedroom as the person with DS.^21^ Caregivers who reported at least moderate sleep disturbances of the person with DS also had higher HADS anxiety scores.^21^

##### Fatigue

Across seven studies reporting fatigue, prevalence ranged from 71.4% (*n* = 5/7 caregivers reported tiredness)^22^ to 84% (*n* = 63/75 caregivers reported often/always experiencing fatigue).^14^ Caregivers frequently described experiencing physical and mental fatigue or exhaustion, and sleep deprivation, often exacerbated by the need for constant supervision and nighttime monitoring of people with DS.^15^, ^16^, ^24^, ^27^ In a large cross‐sectional US study, several significant risk factors for caregiver fatigue included characteristics of the children with rare epilepsy syndromes such as having frequent nighttime awakenings, excess daytime sleepiness, difficulty falling asleep, and very restless sleep; other risk factors included nighttime monitoring methods such as sharing a bed or room with the child, use of an audio monitor or seizure alert device, leaving the door open, and watching or checking frequently.^16^ Caregiver PROMIS depression *T* scores > 60 and caregiver PROMIS anxiety *T* scores > 60 were significantly associated with caregiver fatigue.^16^ Significant protective factors for caregiver fatigue included caregiver PROMIS cognition *T* scores > 60 and PROMIS companionship *T* scores > 60.^16^

##### Stress

Caregivers reported high levels of stress, with impacts on their own mental health.^24^ In a Japanese study, 53% of caregivers of people with DS were reported to have experienced stress.^19^ A multinational study reported that 89% of parents affirmed the need for support for the management of ongoing stress associated with caring for their child with DS.^26^ A Polish study reported that 38.7% of caregivers answered “often” or “always” regarding feeling stressed because of caregiving, with a higher percentage (46.7%) answering “often” or “always” to feeling like they were not coping with stress.^15^

##### Isolation

Four included studies reported isolation,^4^, ^15^, ^17^, ^20^ three of which also reported mental health outcomes of depression and/or anxiety.^4^, ^17^, ^20^ A Polish study reported that 41.3% of caregivers answered “often” or “always” regarding feelings of solitude and isolation as a result of caregiving.^15^ In separate studies where caregivers listed the top three concerns for the patient or family after seizure control, the grouping of anxiety/depression/isolation was among the most common caregiver concerns for two (5.2%) caregivers in a cross‐sectional survey in Taiwan^17^ and in 19 caregivers (no percentage stated) belonging to the Dravet Syndrome Foundation (DSF) Support Group.^4^ A cross‐sectional, interview‐based Japanese study reported accounts from the caregivers' perspective.^20^ One caregiver expressed the emotional burden they felt around the time of onset/diagnosis, describing social isolation as a manifestation of depression. They had to constantly watch over the patient, spent a lot of time at home, and were unable to work. As it was difficult to go out, the caregiver had fewer social interactions and gradually became introverted.

##### Others

Some caregivers reported worry and uncertainty as a result of caregiving,^22^, ^23^ with some referring to emotional states or emotional burden.^15^, ^20^, ^23^, ^25^ One Polish study reported that emotional states of helplessness, nervousness/impulsivity, and hopelessness were experienced “often” or “always” by 72%, 50.7%, and 45.3% of caregivers, respectively.^15^ The emotional impact of caregiving was described in a Spanish study that reported the emotional state subscale of the Childhood Rare Epilepsy Social Impact Assessment psychological domain was significantly higher (indicating a higher impact on caregivers' emotional state) for caregivers of people with DS compared with caregivers of minors without any diagnosed disease.^25^ Another Spanish study reported that 76.1% of caregivers experienced changes in their mood.^13^ A qualitative Japanese study reporting on the emotional journey of caregivers of people with DS highlighted some of the prevailing emotions depending on the stage of the disease journey, with anxiety reported at the time of diagnosis and in regard to the future, and postdiagnosis many caregivers felt depressed when they had to resign from their jobs.^20^ A 2011 multinational study reported that 86% of parents acknowledged experiencing grief regarding their child's condition, and they expressed concern that physicians rarely discuss with their patient's families the possibility of death from SUDEP or status epilepticus.^26^ In a cross‐sectional study of caregivers from the DSF Support Group, 21 caregivers (no percentage stated) reported SUDEP or death as one of their top three concerns for the patient or family after seizure control.^4^ Despite almost one quarter of caregivers in a Spanish study reporting “a lot” of mental health problems in the CarerQoL‐7D questionnaire, 90% of caregivers felt “some” or “a lot” of fulfillment from caregiving.^30^

### 3.4 Limitations of included studies

A total of six studies did not report caregiver characteristics,^12^, ^13^, ^17^, ^18^, ^26^, ^30^ which represents a significant limitation in study design, as understanding caregiver demographics and roles is essential for accurately assessing caregiver burden and its impact on outcomes (Table S5). A small sample size (≤30 caregivers of people with DS) was reported in six studies^12^, ^19^, ^20^, ^22^, ^23^, ^24^ and is expected for studies of rare conditions. However, small sample sizes may reduce the generalizability of the findings and limit the statistical power of the analyses. One study was a single‐center study,^12^ and 12 articles did not report single‐ or multicenter design. Some studies lacked control groups, limiting comparison of the experiences between caregivers of children with DS and those of caregivers of children with other conditions.^22^, ^30^ There was potential recruitment bias, where caregivers who were experiencing more severe symptoms or challenges may have been more likely to participate.^24^ Characteristics of people with DS were not reported for one study,^26^ and some studies did not disclose the upper age limit of people with DS^18^, ^19^, ^25^, ^30^ or the age range limits for children with DS.^12^, ^16^, ^25^, ^26^, ^27^ Some studies used nonvalidated tools/instruments or generic questionnaires to assess mental health and quality of life, which may not capture the specific challenges faced by caregivers of people with DS.

Most caregivers in the majority of studies were female. An SLR of 52 articles from three German cohorts reported that mental health (with depressed mood or symptoms) was worse for women than men, and that depression was more likely to be present among women.^31^ Therefore, experiences of female caregivers may not reflect that of male caregivers and could, thus, limit the applicability of results to male caregivers.

Some studies were undertaken during the coronavirus disease 2019 (COVID‐19) pandemic (declared a global pandemic on March 11, 2020).^32^ The COVID‐19 pandemic has been reported to have impacted the mental health of family caregivers, specifically related to increased levels of anxiety and depression.^33^, ^34^ As such, the COVID‐19 pandemic may have affected the results of some of the studies included in this SLR.^13^, ^19^, ^24^ Some of the included studies were not limited to caregivers of people with DS; one study also included caregivers of people with Lennox–Gastaut syndrome and tuberous sclerosis complex,^20^ and one study included caregivers of people with a variety of rare epilepsy syndromes.^16^

Findings from multinational studies^4^, ^23^, ^26^ might be influenced by cultural and health care system differences, limiting the applicability of results to all caregiver populations. Although this SLR consolidates data from published studies undertaken in different countries, most were undertaken in Europe; there were no published studies from Africa. Outcomes may differ globally owing to regional customs/cultures/differences regarding mental health and health care systems, and there may be cultural differences regarding stigma associated with mental health.

## 4 DISCUSSION

Caregivers of people with DS consistently report significant mental health struggles, including anxiety and depression.^35^ This SLR, highlighting the mental health impacts experienced by caregivers of people with DS, found that only 20 studies fulfilled the inclusion criteria, emphasizing the need for further assessment.

The prevalence of depression and anxiety among caregivers varied widely and may reflect country/cultural‐related differences, restricted age groups of patients with DS (e.g., only ≤18 years of age),^14^, ^15^, ^22^, ^23^ and large differences in sample sizes (ranging from seven in Nabbout et al.^22^ to 256 in Villas et al.^4^).

Stigmas associated with mental health are pervasive and complex issues that impact individuals and communities globally.^36^ Most studies in this SLR were European,^13^, ^14^, ^15^, ^18^, ^21^, ^22^, ^24^, ^25^, ^27^, ^28^, ^29^, ^30^ with few Asian,^17^, ^19^, ^20^ North American,^12^, ^16^ and multinational studies.^4^, ^23^, ^26^ Mental health outcomes of caregivers may have been impacted by regional customs and/or cultures as well as differences in health care systems. A study that used data from the Stigma in Global Context–Mental Health Study reported higher levels of stigma regarding mental health in Eastern countries than in Western countries.^37^ However, data interpretation should be tempered, as caregivers may have felt uncomfortable speaking about their mental health experiences because of the associated stigma and depending on their country of residence and/or cultural background. Stigma associated with mental health is twofold; caregivers may avoid or delay seeking support for their mental health because of the associated stigma,^38^ which in turn may further exacerbate mental health issues. Addressing mental health stigma as part of early support provided to caregivers of people with DS may help to improve mental health outcomes.

In most of the included studies, the mental health burden of caregiving for children with DS was found to predominantly fall on women,^13^, ^14^, ^15^, ^18^, ^19^, ^20^, ^22^, ^23^, ^24^, ^26^ particularly mothers. This highlights the importance of considering gender when designing support programs and interventions.

Several studies revealed a clear association between fatigue, sleep quality, anxiety, and depression, although directionality cannot be assigned. For instance, one study showed that caregivers who reported elevated levels of both anxiety and depression were significantly more likely to have higher fatigue levels.^16^ Similarly, a separate study found that caregivers with higher levels of anxiety were significantly more likely to report at least moderate sleep disturbances.^21^

Emotional well‐being was significantly affected by caregiving for people with DS. One study reported that 76% of caregivers experienced mood changes.^13^ Other studies indicated feelings of helplessness, nervousness/impulsivity, and hopelessness among caregivers.^14^, ^15^ These findings suggest that supporting emotional well‐being is essential in caregiver intervention programs.

Significant protective factors were identified in a study that reported caregivers with higher levels of cognition and perceived companionship were significantly more likely to have lower levels of fatigue and better sleep quality.^16^ Per the PROMIS companionship short form used in the study, companionship is defined as “perceived availability of someone with whom to share enjoyable social activities such as visiting, talking, celebrations, etc.”^39^ Greater social support, including high‐quality relationships, is significantly associated with improved sleep outcomes^40^, ^41^, ^42^ as is participating in social/leisure activities.^43^, ^44^, ^45^ This finding highlights the need to ensure caregivers have access to high‐quality support services, including encouraging caregivers to maintain healthy personal relationships with family and friends to ensure they get the respite needed to safeguard their well‐being.

No studies assessed the impact of SUDEP on the mental health of caregivers. Although one study suggested that unpredictability and worry of SUDEP and nocturnal seizures affect caregivers' sleep patterns (based on the findings that pediatric nocturnal seizures were associated with caregiver sleep disturbance),^16^ and two studies mentioned caregiver concern regarding SUDEP,^4^, ^26^ these studies failed to make a direct association between the concept of SUDEP and any specific mental health outcomes. However, it should be noted that the approach to SUDEP counseling has since evolved.^46^, ^47^ Given the incidence of SUDEP has been reported to be higher among people with DS compared with the general population of people with epilepsy,^26^ and is documented as the most common cause of death among people with DS,^48^, ^49^, ^50^ the impact it has on the mental health of caregivers warrants further investigation.

A number of factors complicate data interpretation. Age limits of people with DS were not clearly reported in some of the included studies, which prevented interpretation of how the age of the person with DS receiving care might impact caregiver mental health, further highlighting the importance of collecting and reporting comprehensive demographic data for people with DS as well as their caregivers. There are also limited data on the number of people with DS who reside at home full‐time or who reside within a nursing home or a specialized facility for people with disabilities. The latter will undoubtedly impact results, as people with DS who reside away from the home will likely be cared for predominantly by employed health care professionals, potentially allowing more respite to family caregivers, which may positively affect the mental health burden of unpaid caregivers.

For studies reported in this SLR that used the BDI‐II^18^, ^28^, ^29^ and/or HADS^19^, ^21^ instruments, the levels of a caregiver's anxiety and depression over the past 1–2 weeks may not be generalizable to wider time frames or to all caregivers. Results will likely differ over time and may also depend on factors such as age and level of need of the person with DS being cared for, the level of support caregivers receive, and the amount of time spent caregiving. Additionally, given the diverse clinical manifestations of DS and the range of severity and seizure types,^7^ caregivers may feel less or more depressed and/or anxious depending on their experience during the previous caregiving week. Because seizure type and frequency as well as nonseizure manifestations change over time,^51^ a better understanding of the DS disease course over time could provide more tailored support to caregivers depending on the age of the person receiving care.

The use of depression and/or anxiety instruments when used alone may predispose authors to focus on these conditions and report on little else. Subjective measures have been used to assess mental health outcomes in caregivers, and few studies have implemented analyses to identify associations between outcomes.^13^, ^16^, ^21^ However, subjective assessments may suggest causality/correlation, which may not be upheld using objective measures, and should be interpreted with caution. Future studies combining both quantitative and qualitative methodologies may enable deeper exploration into the complexities of mental health and may capture additional outcomes that validated instruments alone cannot provide. Incorporation of objective measures could also provide valuable behavioral‐related data, for example, through implementation of polysomnography studies or use of wearable devices to assess sleep quality. Depression and anxiety were reported as symptoms rather than as clinical diagnoses. Future studies should seek to understand what interventions might help caregivers of people with DS with their mental health and whether symptom severity rises to a level requiring treatment.

The absence of a standardized, disease‐specific tool for evaluating caregiver mental health limits consistency and comparability of findings and may overlook key aspects of the caregiving experience specific to DS. This highlights the need for the development and use of validated, standardized instruments tailored to evaluate the unique emotional and psychological challenges faced by caregivers of people with complex, rare conditions such as DS. The development of such tools should be considered a priority for future research.

This SLR is limited by the nature of the data reported across studies. The data were heterogenous between studies, whether qualitative or quantitative in nature. Only studies indexed in Embase or MEDLINE in Ovid were included. Other databases (CINAHL, PsychINFO, Scopus, Web of Science, Cochrane, Google Scholar, Emcare, etc.) might have provided additional records. However, this was a comprehensive SLR conducted using robust methodology and in accordance with PRISMA guidelines, which included both qualitative and quantitative data from publications not restricted by language, time, or country.

Psychosocial interventions for caregivers of people with DS should focus on improving sleep quality, fatigue, and stress and supporting emotional well‐being through strategies such as respite care and social support. Correlating mental health outcomes with one another—or with additional factors such as the age of the person receiving care, amount of time spent caregiving over a set period, and other confounding psychological issues such as fatigue, sleep impairment, and social isolation—could provide insight into which factors have the biggest impact on mental health and which could be best targeted for support. It is likely that bidirectional relationships between mental health outcomes exist; therefore, a more holistic approach is needed to provide support for the mental health of caregivers of people with DS.

The challenges identified in this SLR highlight the urgent need for improvement on the part of primary care providers and physicians to enquire about the mental health of caregivers. Current practice should adopt systems whereby physicians routinely and proactively assess the mental health of caregivers. The importance of patient advocacy groups (PAGs) should also be highlighted, as reflected by the Caregiver Insight Survey, which reported 71% of caregivers changed neurologists/epileptologists most commonly because of clinical expertise and trust, often seeking information from trusted PAGs.^52^ A recent Delphi consensus reported strong agreement for parents/caregivers being given the contact details of DS PAGs, along with DS‐specific materials (brochures/guides/websites) at the end of the first consultation.^53^ Despite these recommendations, little is reported on the effects of PAG support on the mental health of caregivers of people with DS.

## 5 CONCLUSIONS

Overall, this SLR provides a comprehensive and up‐to‐date overview of the mental health impacts experienced by caregivers of people with DS. Caregivers of people with DS consistently report significant mental health struggles, including mental health conditions such as depression and anxiety, and factors potentially associated with mental health such as poor sleep quality, fatigue, stress, and isolation. Additional studies on caregivers of people with DS are required to further assess the impact of caregiving on mental health. There is a need for validated, standardized instruments tailored to assess emotional and psychological challenges experienced by caregivers of people with DS, along with identification of interventions and support systems that might help ease caregiver burden.

## AUTHOR CONTRIBUTIONS

**Adam Strzelczyk:** Methodology; investigation; visualization; writing of the original draft; reviewing and editing the manuscript. **Mary Anne Meskis:** Methodology; investigation; visualization; writing of the original draft; reviewing and editing the manuscript. **Galia Wilson:** Methodology; investigation; visualization; writing of the original draft; reviewing and editing the manuscript. **Bobby Jacob:** Conceptualization; funding acquisition; methodology; investigation; project administration; resources; supervision; validation; visualization; writing of the original draft; reviewing and editing the manuscript. **Christoph Helmstaedter:** Methodology; investigation; visualization; writing of the original draft; reviewing and editing the manuscript. **Jane von Gaudecker:** Methodology; investigation; visualization; writing of the original draft; reviewing and editing the manuscript. **Veronica Hood:** Methodology; investigation; visualization; writing of the original draft; reviewing and editing the manuscript. **Ceri Hughes:** Investigation; visualization; writing of the original draft; reviewing and editing the manuscript. **Michael Scott Perry:** Methodology; investigation; visualization; writing of the original draft; reviewing and editing the manuscript.

## FUNDING INFORMATION

This study was funded by UCB, which was involved in the design of the systematic literature review; in the collection, analysis, and interpretation of data; and in the decision to publish the article.

## CONFLICT OF INTEREST STATEMENT

A.S. has received personal fees and grants from Angelini Pharma, Biocodex, Desitin Arzneimittel, Eisai, Longboard, Jazz Pharmaceuticals, Neuraxpharm, Stoke Therapeutics, Takeda, UCB Pharma, and UNEEG medical. M.A.M. is the chief executive officer of the Dravet Syndrome Foundation. G.W. is the chair of trustees at Dravet Syndrome UK. B.J. is an employee of UCB. C.He. receives personal fees and support from Angelini Pharma, Desitin Arzneimittel, Eisai, Jazz Pharmaceuticals, and UCB; license fees from Eisai and UCB (Japan); grant funds from University Bonn; honoraria from the National Center for Epilepsy (Oslo, Norway) and the Florey (Melbourne, Australia); and is an associate editor of *Seizure*. J.v.G. has no conflicts of interest to disclose. V.H. is chief scientific officer at the Dravet Syndrome Foundation. C.Hu. is chief science officer at Dravet Syndrome UK. M.S.P. serves as a consultant for Azurity, Marinus, Neurelis, Pyros, Stoke Therapeutics, UCB, and Jazz Pharmaceuticals and holds unpaid board memberships with the Child Neurology Foundation, Dravet Syndrome Foundation, Lennox–Gastaut Foundation, and Pediatric Epilepsy Research Consortium; research funding has been provided to M.S.P.'s institution by Biocodex, Encoded, Neurocrine, Stoke, Takeda, and UCB. We confirm that we have read the Journal's position on issues involved in ethical publication and affirm that this report is consistent with those guidelines.

## Supporting information


TABLES S1–S5.

## Data Availability

Data from noninterventional studies are outside of UCB's data sharing policy and are unavailable for sharing.
