# Supplementary material for: Mental health impacts experienced by caregivers of people with Dravet syndrome: A systematic literature review
Source: Epilepsia. 2026 Mar 30;67(6):3082–107. doi: 10.1002/epi.70171 (PMC13285260; doi:10.1002/epi.70171)
Supplement: Supplementary file 1 — TABLES S1–S5. [file EPI-67-3082-s001.docx]

SUPPLEMENTARY INFORMATION

| **TABLE S1.** Keywords searched in Embase and MEDLINE. | | |
| --- | --- | --- |
| **Searches in Embase (1974 to August 29, 2024)** | | **Results** |
| 1 | exp severe myoclonic epilepsy in infancy/ | 3065 |
| 2 | (Dravet syndrome or SMEI).mp. | 2800 |
| 3 | 1 or 2 | 3781 |
| 4 | exp caregiver/ or exp caregiver burden/ or exp caregiver support/ or exp mother/ or exp father/ or exp parent/ or exp family/ or ((carer or care) adj2 provider).mp. | 762,230 |
| 5 | exp mental health/ or exp depression/ or exp anxiety/ | 1,052,550 |
| 6 | (stress or burden or impact or cope or coping or dysfunction or challenge or function or concern or difficult* or mood or psychosocial or psycho?social or psycholog* or social* or emotion* or wellbeing or well-being or trauma* or panic or mental).mp. | 12,647,696 |
| 7 | exp "quality of life"/ or exp psychological aspect/ or exp psychological adjustment/ or exp psychological well-being/ | 1,215,457 |
| 8 | 5 or 6 or 7 | 13,106,392 |
| 9 | 3 and 4 | 355 |
| **Searches in Ovid MEDLINE ALL (1946 to August 29, 2024)** | | |
| 1 | exp Epilepsies, Myoclonic/ | 5629 |
| 2 | (Dravet syndrome or SMEI).mp. | 1708 |
| 3 | 1 or 2 | 6347 |
| 4 | exp caregiver/ or exp caregiver burden/ or exp caregiver support/ or exp mother/ or exp father/ or exp parent/ or exp family/ or ((carer or care) adj2 provider).mp. | 397,333 |
| 5 | exp mental health/ or exp depression/ or exp anxiety/ | 298,766 |
| 6 | (stress or burden or impact or cope or coping or dysfunction or challenge or function or concern or difficult* or mood or psychosocial or psycho?social or psycholog* or social* or emotion* or wellbeing or well-being or trauma* or panic or mental).mp. | 9,299,994 |
| 7 | exp "quality of life"/ or exp psychological aspect/ or exp psychological adjustment/ or exp psychological well-being/ | 295,244 |
| 8 | 5 or 6 or 7 | 9,426,522 |
| 9 | 3 and 4 | 164 |

*Note*: exp = explodes indexing term: expands search to all more specific related terms in the vocabulary's hierarchy; .mp. = combined search fields (default if no fields are specified).

Abbreviation: SMEI, severe myoclonic epilepsy in infancy.

| TABLE S2. Part I: Mixed Methods Appraisal Tool (MMAT), version 2018. | | | | | |
| --- | --- | --- | --- | --- | --- |
| Category of study designs | Methodological quality criteria | Responses | | | |
|  |  | Yes | No | Can’t tell | Comments |
| Screening questions  (for all types) | S1. Are there clear research questions? |  |  |  |  |
|  | S2. Do the collected data allow to address the research questions? |  |  |  |  |
|  | Further appraisal may not be feasible or appropriate when the answer is “No” or “Can’t tell” to one of both screening questions. | | | | |
| 1. Qualitative | 1.1. Is the qualitative approach appropriate to answer the research question? |  |  |  |  |
|  | 1.2. Are the qualitative data collection methods adequate to address the research question? |  |  |  |  |
|  | 1.3. Are the findings adequately derived from eth data? |  |  |  |  |
|  | 1.4. Is the interpretation of results sufficiently substantiated by data? |  |  |  |  |
|  | 1.5. Is there coherence between qualitative data sources, collection, analysis and interpretation? |  |  |  |  |
| 1. Quantitative randomized controlled trials | 2.1. Is randomization appropriately performed? |  |  |  |  |
|  | 2.2. Are the groups comparable at baseline? |  |  |  |  |
|  | 2.3. Are there complete outcome data? |  |  |  |  |
|  | 2.4. Are outcome assessors blinded to the intervention provided? |  |  |  |  |
|  | 2.5. Did the participants adhere to the assigned intervention? |  |  |  |  |
| 1. Quantitative  non-randomized | 3.1. Are the participants representative of the target population? |  |  |  |  |
|  | 3.2. Are measurements appropriate regarding both the outcome and intervention (or exposure)? |  |  |  |  |
|  | 3.3. Are there complete outcome data? |  |  |  |  |
|  | 3.4. Are the confounders accounted for in the design and analysis? |  |  |  |  |
|  | 3.5. During the study period, is the intervention administered (or exposure occurred) as intended? |  |  |  |  |
| 1. Quantitative descriptive | 4.1. Is the sampling strategy relevant to address the research question? |  |  |  |  |
|  | 4.2. Is the sample representative of the target population? |  |  |  |  |
|  | 4.3. Are measurements appropriate? |  |  |  |  |
|  | 4.4 Is the risk of nonresponse bias low? |  |  |  |  |
|  | 4.5. Is the statistical analysis appropriate to answer the research question? |  |  |  |  |
| 1. Mixed methods | 5.1. Is there an adequate rationale for using mixed methods design to address the research question? |  |  |  |  |
|  | 5.2. Are the different components of the study effectively integrated to answer the research question? |  |  |  |  |
|  | 5.3. Are the outputs of the integration of qualitative and quantitative components adequately integrated? |  |  |  |  |
|  | 5.4. Are divergences and inconsistencies between quantitative and qualitative results adequately addressed? |  |  |  |  |
|  | 5.5 Do the different components of the study adhere to the quality criteria of each tradition of the methods involved? |  |  |  |  |
| *Note*: Table adapted from Hong QN, et al. Mixed Methods Appraisal Tool (MMAT), version 2018. Registration of Copyright (#1148552), Canadian Intellectual Property Office, Industry Canada.[^11^](#_ENREF_11) | | | | | |

| **TABLE S3.** Articles excluded at the full-text screening step. | |
| --- | --- |
| **Reference** | **Reason for exclusion** |
| Aledo-Serrano A, Mingorance A. Analysis of the family impact and needs of Dravet's syndrome in Spain. [Spanish] Rev Neurol. 2020;70(3):75-83. | No mental health outcomes |
| Boyce DM, Devinsky O, Meskis MA. Barriers to transition from pediatric to adult care for patients with Dravet syndrome: a focus group study of caregivers. Epilepsy Behav. 2020;109:107096. | No mental health outcomes |
| Camfield P, Camfield C, Nolan K. Helping families cope with the devastation of Dravet syndrome. [Review] Eur J Paediatr Neurol. 2012;16(Suppl 1):S9-12. | Review |
| Camfield P, Camfield C, Nolan K. Helping families cope with the severe stress of Dravet syndrome. Can J Neurol Sci. 2016;43(Suppl 3):S9-S12. | Review |
| Carter B, Cook G, Bray L, Collingwood A, Saron H, Rouncefield-Swales A. Parents'/caregivers' fears and concerns about their child's epilepsy: a scoping review. PLoS ONE. 2022;17(9):e0274001. | Review |
| Ferretti A, Riva A, Fabrizio A, Bruni O, Capovilla G, Foiadelli T, Orsini A, Raucci U, Romeo A, Striano P, Parisi P. Best practices for the management of febrile seizures in children. Ital J Pediatr. 2024;50(1):95. | Review |
| Gonçalves C, Martins S, Fernandes L. Dravet syndrome: effects on informal caregivers' mental health and quality of life - A systematic review. Epilepsy Behav. 2021;122:108206. | Review |
| Jensen MP, Brunklaus A, Dorris L, Zuberi SM, Knupp KG, Galer BS, Gammaitoni AR. The humanistic and economic burden of Dravet syndrome on caregivers and families: implications for future research. Epilepsy Behav. 2017;70(Pt A):104-9. | Review |
| Jensen MP, Gammaitoni AR, Galer BS, Salem R, Wilkie D, Amtmann D. Fenfluramine treatment for Dravet syndrome: real-world benefits on quality of life from the caregiver perspective. Epilepsy Res. 2022;185:106976. | Pharmacological interventions |
| Jensen MP, Gammaitoni AR, Salem R, Wilkie D, Lothe A, Amtmann D. Fenfluramine treatment for Dravet syndrome: caregiver- and clinician-reported benefits on the quality of life of patients, caregivers, and families living in Germany, Spain, Italy, and the United Kingdom. Epilepsy Res. 2023;190:107091. | Pharmacological interventions |
| Juando-Prats C, James E, Bilder DA, McNair L, Kenneally N, Helfer J, Huang N, Vila MC, Sullivan J, Wirrell E, Rico S. DRAVET ENGAGE. Parent caregivers of children with Dravet syndrome: perspectives, needs, and opportunities for clinical research. Epilepsy Behav. 2021;122:108198. | No mental health outcomes |
| Lagae L, Irwin J, Gibson E, Battersby A. Caregiver impact and health service use in high and low severity Dravet syndrome: a multinational cohort study. Seizure. 2019;65:72-9. | No mental health outcomes |
| Lo SH, Lloyd A, Marshall J, Vyas K. Patient and caregiver health state utilities in Lennox-Gastaut syndrome and Dravet syndrome. Clin Ther. 2021;43(11):1861-1876.e16. | No mental health outcomes |
| Nabbout R, Dirani M, Teng T, Bianic F, Martin M, Holland R, Chemaly N, Coque N. Impact of childhood Dravet syndrome on care givers of patients with DS, a major impact on mothers. Epilepsy Behav. 2020;108:107094. | No mental health outcomes |
| Nolan K, Camfield CS, Camfield PR. Coping with a child with Dravet syndrome: insights from families. J Child Neurol. 2008;23(6):690-4. | No mental health outcomes |
| Nolan KJ, Camfield CS, Camfield PR. Coping with Dravet syndrome: parental experiences with a catastrophic epilepsy. Dev Med Child Neurol. 2006;48(9):761-5. | No mental health outcomes |
| Paprocka J, Lewandowska A, Zieliński P, Kurczab B, Emich-Widera E, Mazurczak T. Dravet syndrome —The Polish Family's Perspective Study. J Clin Med. 2021;10(9):1903. | No caregiver outcomes |
| Pinsent A, Weston G, Adams EJ, Linley W, Hawkins N, Schwenkglenks M, Hamlyn-Williams C, Toward T. Determining the relationship between seizure-free days and other predictors of quality of life in patients with Dravet syndrome and their carers from FFA registration studies. Neurol Ther. 2023;12(5):1593-606. | No mental health outcomes |
| Soto Jansson J, Bjurulf B, Dellenmark Blom M, Hallböök T, Reilly C. Diagnosis, epilepsy treatment and supports for neurodevelopment in children with Dravet syndrome: caregiver reported experiences and needs. Epilepsy Behav. 2024;151:109603. | No mental health outcomes |
| Strzelczyk A, Lagae L, Kurlemann G, Flege S, Bast T, Polster T, Pringsheim M, von Spiczak S, Hipp P, Schubert-Bast S. Clinical characteristics and quality of life with Dravet syndrome: results of the German cohort of the Dravet syndrome caregiver survey (DISCUSS). [German] Monatsschrift fur Kinderheilkunde. 2024;172(7):606-14. | No mental health outcomes |

| **TABLE S4.** Quality appraisal of included publications using the Mixed Methods Appraisal Tool. | | | | | | | |
| --- | --- | --- | --- | --- | --- | --- | --- |
| **Qualitative studies** | | | | | | | |
| **Publication** | **Are there clear research questions?** | **Do the collected data allow to address the research questions?** | **Is the qualitative approach appropriate to answer the research question?** | **Are the qualitative data collection methods adequate to address the research question?** | **Are the findings adequately derived from the data?** | **Is the interpretation of results sufficiently substantiated by data?** | **Is there coherence between qualitative data sources, collection, analysis and interpretation?** |
| Nabbout R, Auvin S, Chiron C, Irwin J, Mistry A, Bonner N, Williamson N, Bennett B. Development and content validation of a preliminary core set of patient- and caregiver-relevant outcomes for inclusion in a potential composite endpoint for Dravet syndrome.  Epilepsy Behav. 2018:78:232-42. | Yes | Yes | Yes | Yes | Yes | Yes | Yes |
| Nabbout R, Auvin S, Chiron C, Thiele E, Cross H, Scheffer IE, Schneider AL, Guerrini R, Williamson N, Zogenix and Adelphi Values study group. Perception of impact of Dravet syndrome on children and caregivers in multiple countries: looking beyond seizures.  Dev Med Child Neurol. 2019;61(10):1229-36. | Yes | Yes | Yes | Yes | Yes | Yes | Yes |
| Postma A, Milota M, Jongmans MJ, Brilstra EH, Zinkstok JR. Challenging behavior in children and adolescents with Dravet syndrome: exploring the lived experiences of parents*.* Epilepsy Behav. 2023:138:108978. | Yes | Yes | Yes | Yes | Yes | Yes | Yes |
| Soto Jansson J, Bjurulf B, Dellenmark Blom M, Hallböök T, Reilly C. Caregiver perceptions of the impact of Dravet syndrome on the family, current supports and hopes and fears for the future: a qualitative study. Epilepsy Behav. 2024:156:109790. | Yes | Yes | Yes | Yes | Yes | Yes | Unclear |
| **Quantitative non-randomized trials** | | | | | | | |
| **Publication** | **Are there clear research questions?** | **Do the collected data allow to address the research questions?** | **Are the participants representative of the target population?** | **Are measurements appropriate regarding both the outcome and intervention (or exposure)?** | **Are there complete outcome data?** | **Are the confounders accounted for in the design and analysis?** | **During the study period, is the intervention administered (or exposure occurred) as intended?** |
| Campbell JD, Whittington MD, Kim CH, VanderVeen GR, Knupp KG, Gammaitoni A. Assessing the impact of caring for a child with Dravet syndrome: results of a caregiver survey.  Epilepsy Behav. 2018:80:152-6. | Yes | Yes | Unclear | Yes | Yes | Unclear | Yes |
| Hesdorffer DC, Kroner BL, Shen J, Farrell K, Roberds S, Fureman B. Factors associated with caregiver sleep quality related to children with rare epilepsy syndromes. J Pediatr: X. 2020;2:100021. | Yes | Yes | Unclear | Yes | Yes | Yes | Yes |
| Kalski M, Schubert-Bast S, Kieslich M, Leyer A-C, Polster T, Herting A, Mayer T, Trollmann R, Neubauer BA, Bettendorf U, Bast T, Wiemer-Kruel A, von Spiczak S, Kurlemann G, Wolff M, Kluger G, Carroll J, Macdonald D, Pritchard C, Irwin J, Klein KM, Rosenow F, Strzelczyk A, Kay L. Clinical characteristics, resource utilization, quality of life and care situation for patients with Dravet syndrome in Germany. Z. Epileptol. 2019:32:326-38. | Yes | Yes | Unclear | Yes | Yes | No | Yes |
| LoPresti M, Igarashi A, Sonohara Y, Bowditch S. A quantitative cross-sectional study of the burden of caring for patients with Lennox-Gastaut syndrome, Dravet syndrome, and tuberous sclerosis complex-associated epilepsy in Japan. Epilepsy Behav. 2024:154:109741. | Yes | Yes | Unclear | Yes | Yes | Unclear | Yes |
| Maltseva M, Schubert-Bast S, Zöllner JP, Bast T, Mayer T, von Spiczak S, Ruf S, Trollmann R, Wolff M, Hornemann F, Klotz KA, Jacobs J, Kurlemann G, Neubauer BA, Polster T, Syrbe S, Bertsche A, Bettendorf U, Kluger G, Flege S, Rosenow F, Kay L, Strzelczyk A. Sleep quality, anxiety, symptoms of depression, and caregiver burden among those caring for patients with Dravet syndrome: a prospective multicenter study in Germany. Orphanet J Rare Dis. 2023;18(1):98. | No | Unclear | Unclear | Unclear | Yes | No | Yes |
| Salom R, Aras LM, Piñero J, Duñabeitia JA. The psychosocial impact of caring for children with Dravet syndrome*.* Epilepsy Behav Rep. 2023;24:100619. | Yes | Yes | Unclear | Yes | Yes | No | Yes |
| Strzelczyk A, Schubert-Bast S, Bast T, Bettendorf U, Fiedler B, Hamer HM, Herting A, Kalski M, Kay L, Kieslich M, Klein KM, Kluger G, Kurlemann G, Mayer T, Neubauer BA, Polster T, von Spiczak S, Stephani U, Trollmann R, Wiemer-Kruel A, Wolff M, Irwin J, Carroll J, Pritchard C, Rosenow F. A multicenter, matched case-control analysis comparing burden-of-illness in Dravet syndrome to refractory epilepsy and seizure remission in patients and caregivers in Germany. Epilepsia. 2019;60(8):1697-710. | Yes | Yes | Unclear | Yes | Yes | Yes | Yes |
| Strzelczyk A, Kalski M, Bast T, Wiemer-Kruel A, Bettendorf U, Kay L, Kieslich M, Kluger G, Kurlemann G, Mayer T, Neubauer BA, Polster T., Herting A., von Spiczak S., Trollmann R., Wolff M., Irwin J., Carroll J, Macdonald D, Pritchard C, Klein KM, Rosenow F, Schubert-Bast S. Burden-of-illness and cost-driving factors in Dravet syndrome patients and carers: a prospective, multicenter study from Germany. Eur J Paediatr Neurol. 2019;23(3):392-403. | No | Unclear | Unclear | Unclear | Yes | Yes | Yes |
| Gil-Nagel A, Sánchez-Carpintero R, Villanueva V. Patient profile, management, and quality of life associated with Dravet syndrome: a cross-sectional, multicentre study of 80 patients in Spain. Sci Rep. 2023;13(1):3355. | Yes | Yes | Yes | Yes | Yes | Yes | Yes |
| **Quantitative descriptive studies** | | | | | | | |
| **Publication** | **Are there clear research questions?** | **Do the collected data allow to address the research questions?** | **Is the sampling strategy relevant to address the research question?** | **Is the sample representative of the target population?** | **Are the measurements appropriate?** | **Is the risk of nonresponse bias low?** | **Is the statistical analysis appropriate to answer the research question?** |
| Cardenal-Muñoz E, Nabbout R, Boronat S, Lara-Herguedas J, Villanueva V, Aibar JÁ. Impact of COVID-19 on Spanish patients with Dravet syndrome and their caregivers: consequences of lockdown. Rev Neurol. 2021;73(2):57-65. | Yes | Yes | No | Unclear | Yes | Unclear | Yes |
| Domaradzki J, Walkowiak D. Caring for children with Dravet syndrome: exploring the daily challenges of family caregivers. Children (Basel). 2023;10(8):1410. | No | Unclear | No | Unclear | Yes | Unclear | Yes |
| Domaradzki J, Walkowiak D. Emotional experiences of family caregivers of children with Dravet syndrome. Epilepsy Behav. 2023:142:109193. | Yes | Yes | No | Unclear | Yes | Unclear | Yes |
| Huang C-H, Hung P-L, Fan P-C, Lin K-L, Hsu T-R, Chou I-J, Ho C-S, Chou I-C, Lin W-S, Lee I-C, Fan H-C, Chen S-J, Liang J-S, Tu Y-F, Chang T-M, Hu S-C, Wong L-C, Hung K‑L, Lee W-T. Clinical spectrum and the comorbidities of Dravet syndrome in Taiwan and the possible molecular mechanisms. Sci Rep. 2021;11(1):20242. | Yes | Yes | No | Unclear | Yes | Unclear | Yes |
| Skluzacek JV, Watts KP, Parsy O, Wical B, Camfield P. Dravet syndrome and parent associations: The IDEA League experience with comorbid conditions, mortality, management, adaptation, and grief. Epilepsia. 2011:52(Suppl 2):95-101. | No | Unclear | No | Unclear | Yes | Unclear | Yes |
| Villas N, Meskis MA, Goodliffe S. Dravet syndrome: characteristics, comorbidities, and caregiver concerns. Epilepsy Behav. 2017:74:81-6. | Yes | Yes | No | Unclear | Yes | Unclear | Yes |
| **Mixed methods studies** | | | | | | | |
| **Publication** | **Are there clear research questions?** | **Do the collected data allow to address the research questions?** | **Is there an adequate rationale for using a mixed methods design to address the research question?** | **Are the different components of the study effectively integrated to answer the research question?** | **Are the outputs of the integration of qualitative and quantitative components adequately interpreted?** | **Are divergences and inconsistencies between quantitative and qualitative results adequately addressed?** | **Do the different components of the study adhere to the quality criteria of each tradition of the methods involved?** |
| LoPresti M, Igarashi A, Sonohara Y, Bowditch S. The emotional burden of caring for patients with Lennox-Gastaut syndrome, Dravet syndrome, and tuberous sclerosis complex-associated epilepsy: a qualitative study in Japan. Epilepsy Behav. 2024:158:109932. | Yes | Yes | Yes | Yes | Yes | Yes | Yes |
| *Note*: MMAT questions from Mixed Methods Appraisal Tool (MMAT), version 2018. Registration of Copyright (#1148552), Canadian Intellectual Property Office, Industry Canada.[^11^](#_ENREF_11)  Abbreviation: MMAT, Mixed Methods Appraisal Tool. | | | | | | | |

##

| **TABLE S5.** Limitations of included articles. | | | | |
| --- | --- | --- | --- | --- |
| **Author, year Country** | **Small sample size (≤30** **caregivers of people with DS**) | **Single-center study** | **Use of only caregiver surveys (instead of/ as well as instruments) to measure components of mental health** | **Other limitations** |
| **Campbell, 2018**[**^12^**](#_ENREF_12) **USA** | Yes | Yes | No | - Recall and response biases may exist as collected data were reliant on the recollections and impressions of caregivers |
| **Cardenal-Muñoz, 2021**[**^13^**](#_ENREF_13) **Spain** | No | NR | Yes | - No caregiver characteristics were reported - The study was conducted during the COVID-19 pandemic; as such, findings may not be representative of usual caregiver experience |
| **Domaradzki, 2023a**[**^14^**](#_ENREF_14) **Poland** | No | NR | Yes | - The results cannot be extrapolated for the entire population of DS caregivers in Poland - The survey used was not validated - No spontaneous reporting because a predefined questionnaire was used - Most participants were women, mainly mothers - The study does not represent experiences of caregivers of adults with DS |
| **Domaradzki, 2023b**[**^15^**](#_ENREF_15) **Poland** | No | NR | Yes | - The results cannot be extrapolated for the entire population of DS caregivers in Poland - The survey used was not validated - No spontaneous reporting because a predefined questionnaire was used - Most participants were women, mainly mothers - The study does not represent experiences of caregivers of adults with DS |
| **Hesdorffer, 2020**[**^16^**](#_ENREF_16) **USA** | No | NR | No | - The results were not specific to caregivers of just children with DS, but also included data from caregivers of children with other epilepsy-related syndromes - The caregiver population comprised mostly White, non-Hispanic females who were highly educated and relatively affluent; as such, findings may not be generalizable to all caregivers of individuals with DS - The study does not represent experiences of caregivers of adults with DS |
| **Huang, 2021**[**^17^**](#_ENREF_17) **Taiwan** | No | NR | Yes | - No caregiver characteristics were reported - Lack of generalizability of findings |
| **Kalski, 2019**[**^18^**](#_ENREF_18) **Germany** | No | No | No | - Possibility of bias resulting from the recruitment approach |
| **LoPresti, 2024a**[**^19^**](#_ENREF_19) **Japan** | Yes | NR | No | - The study included caregivers of patients with rare epilepsy conditions in addition to just DS, including Lennox-Gastaut syndrome and tuberous sclerosis complex-associated epilepsy. However, supplementary tables reported on caregivers of individuals with DS separately - All caregivers were female - Given almost half of caregivers reported that time spent caregiving during the COVID-19 pandemic increased, caregiver HRQoL and emotional wellbeing results may have been impacted by the COVID-19 pandemic |
| **LoPresti, 2024b**[**^20^**](#_ENREF_20) **Japan** | Yes | NR | Yes | - The study included patients with rare epilepsy conditions including Lennox-Gastaut syndrome, DS, and tuberous sclerosis complex-associated epilepsy; as such, findings may not be generalizable to just caregivers of individuals with DS - Most caregivers were female - When this study was conducted, there were no validated disease‑specific instruments to qualitatively assess emotional burden in rare, refractory, and severe childhood-onset epilepsies - Interviewer bias may have been present due to the emotional subject matter, and the interviewer’s aim of ensuring caregivers were comfortable with communicating their opinions |
| **Maltseva, 2023**[**^21^**](#_ENREF_21) **Germany** | No | No | No | - The study was conducted only in Germany - No clinical diagnosis of depression was made in caregivers on the basis of survey data; as such, only symptoms of depression and anxiety were reported - Only the main caregiver completed the questionnaire - Most caregivers were female |
| **Nabbout, 2018**[**^22^**](#_ENREF_22) **France** | Yes | No | Yes | - The interview guide was developed and reviewed by clinical experts, with no involvement from caregivers - Given the lack of a control group (e.g., patients with epilepsy without DS), findings may not be specific to just DS - No adult people with DS were included in interviews; no experiences of caregivers of adults with DS were included |
| **Nabbout, 2019**[**^23^**](#_ENREF_23) **Multinational** | Yes | No | Yes | - Small sample size of caregivers recruited from each country - The interview guide used was developed for the French study,[^22^](#_ENREF_22) which was developed and reviewed by clinical experts , with no involvement from caregivers - Most caregivers were female - Only caregivers of children with DS were interviewed - The results reflect a Western societal perspective on the impact of DS - Differences between countries may result in differences in patient characteristics (e.g., DS severity), caregiver demographic characteristics (e.g., sex, age), and health care systems |
| **Postma, 2023**[**^24^**](#_ENREF_24) **Belgium (Flanders) and The Netherlands** | Yes | NR | Yes | - Caregivers were all female (mothers) - Potential recruitment bias may have been introduced as caregiver participation was based on caregivers contacting the research team after reading either an email or an advertisement on social media - Caregivers who experienced difficulties with their child’s behavior may have been more likely to participate in the study, with results potentially being reflective of the more severe behavior associated with DS - Results may have been impacted by the COVID-19 pandemic |
| **Salom, 2023**[**^25^**](#_ENREF_25) **Spain** | No | NR | No | - Methodology surrounding the recruitment process was not disclosed - Most caregivers were female |
| **Skluzacek, 2011**[**^26^**](#_ENREF_26) **Multinational** | No | NR | Yes | - No patient or caregiver characteristics were reported - Although caregivers (members of the IDEA League) were stated to have lived in 46 countries (most lived in Australia, Canada, the United Kingdom, and the USA), for the 57 parents who answered questions in the 2009 survey regarding grief and adaptation, it is unknown which countries these caregivers resided in at the time of survey participation |
| **Soto Jansson, 2024**[**^27^**](#_ENREF_27) **Sweden** | No | NR | Yes | - Most caregivers were female - Interviews were not all conducted by the same person, and not all were face-to-face - Interviews were analyzed by the individual who conducted the interviews; as such, the researchers conducting the interviews and those involved in the analyses were not independent of one another |
| **Strzelczyk, 2019a**[**^28^**](#_ENREF_28) **Germany** | No | No | No | - The study was conducted only in Germany - No caregiver characteristics were reported - Given the low number of adult patients with DS included in the study, the outcome of the study was pediatric-focused - The initial independent studies from which data were collected for the study were conducted several years apart |
| **Strzelczyk, 2019b**[**^29^**](#_ENREF_29) **Germany** | No | No | No | - No caregiver characteristics were reported |
| **Villas, 2017**[**^4^**](#_ENREF_4) **Multinational** | No | NR | Yes | - Given that most of the survey respondents were from the USA (70%) followed by the United Kingdom (13%), the results reflect a Western societal perspective on the impact of DS - Sources of potential bias may have included:   - caregiver perception and response to the survey;   - the survey was online and only in English;   - caregivers of severely affected individuals may have been overrepresented in the support group; and   - the survey design was based on previous parent-reported surveys and topics of discussion in the Dravet Syndrome Foundation online support group |
| **Gil-Nagel, 2023**[**^30^**](#_ENREF_30) **Spain** | No | No | No | - No caregiver characteristics were reported - The study included only patients who had been followed in referral centers in Spain - No control group was included in the study - A generic and an ad hoc questionnaire was used to evaluate HRQoL in DS owing to the absence of a validated questionnaire |
| Abbreviations: COVID-19, coronavirus disease 2019; DS, Dravet syndrome; HRQoL, Health-related quality of life; IDEA, International DS Epilepsy Action; QoL, quality of life; NR, not reported; USA, Unites States of America. | | | | |
|  | | | | |
